# Lower limb prosthetic users’ activity classification through real-world inertial sensing

**DOI:** 10.1371/journal.pone.0358882

**Published:** 2026-10-09

**Authors:** Matthew Wassall, Malcolm Granat, Saeed Zahedi, Sibylle Thies

**Affiliations:** 1 Department of Neuromedicine and Movement Science, Norwegian University of Science and Technology, Trondheim, Norway; 2 School of Health & Society, University of Salford, Salford, United Kingdom; 3 Blatchford Prosthetics, Basingstoke, United Kingdom; St Mary's University, UNITED STATES OF AMERICA

## Abstract

The prescription of lower limb prosthetics relies heavily on the assigned K-level. K-level assessment has been criticised for subjectivity and variability, potentially leading to inappropriate component selection and poorer functional outcomes. This study presents an original methodology for objectively determining activity levels from inertial sensor data, developed through rigorous data collection on 20 amputees who walked on various types of outdoor terrain (flat ground, stairs, ramp, grass/uneven/unstable surfaces), with and without a walking aid. Advanced machine learning algorithms were applied to the real-world IMU data, to establish a validated classification model for daily activity outside the laboratory. A random forest classifier achieved a mean leave‑some‑out accuracy of 65.70% (SD 6.6%; 95% CI 47.3–84.1%) and cross‑validation accuracy of 85.65% (SD 0.79%; 95% CI 83.4–87.9%). Per‑class sensitivity ranged from approximately 44–78% in leave‑some‑out analyses, with lower performance for ramp ascending and descending. The difference between cross‑validation and leave‑some‑out accuracies indicates participant‑specific overfitting, so the leave‑some‑out accuracy is considered the current conservative estimate of performance on unseen users and the cross-validation accuracy an estimate of potential accuracy with a more robust dataset. The research subsequently incorporated a preliminary clinical evaluation of the approach on a subset of 3 amputees using a data collection protocol for unsupervised, longitudinal real-world monitoring, with preliminary insight into perceived clinical significance of outcomes ascertained through exploratory interviews with 4 prescribing health care professionals. The findings suggest potential benefits of real-world inertial sensing, supporting a move from primarily subjective clinical assessment toward more objective, data-driven activity classification. This research demonstrates that an evidence-based tool for enhancing K-level prescription could in future provide more appropriate component selection and improve long-term mobility for prosthetic users, but current accuracies and sample sizes indicate that the system should be viewed as a promising proof‑of‑concept rather than a definitive tool for changing K‑level prescription.

## Introduction

It is estimated that 35–40 million people globally require prosthetics and orthotics [1], and the need for prosthesis in particular is on the rise, for example in 2005 there were 1.6 million people living with limb loss in the United States of America (USA) and it is projected that there will be 3.6 million by 2050 [2]. Activity levels of lower limb prosthesis patients are characterised by K-levels and the assigned K-level determines which prosthesis components the patient will be given [3]. K-levels were established by the US Health Care Financing Administration and span from K0 to K4 [3]. There is no set procedure to assess patients for classification of K-levels. K-level assessments variability and subjectivity can at times lead to inappropriate prosthetic prescriptions which could impact the user’s functional outcomes [4].

One solution to improve the reliability of K-level assessments could be the use of activity monitoring in the real-world, to provide clinicians with objective every-day data on activities which could support clinicians in making more consistent K‑level assessments and justify component selection, but this relationship has not yet been empirically demonstrated. Activity monitoring involves the use of inertial measurement units (IMUs) placed on the body that measure acceleration, rotation, and orientation. IMUs provide data which can then be used to characterise movements of the body. Activity monitors are increasingly used in human movement science, but they are not yet routinely employed in clinical practice to assess patients’ activity levels [5]. Notably, understanding what measures derived from IMU data could potentially be useful to support K-level assessments is key to supporting clinical decision-making.

As a first step, we conducted exploratory interviews with 6 clinical experts to identify issues with current K-level assessments and what measures would be desirable to help improve the reliability of the assessments [6]. These interviews revealed that the issues with the current assessments relate to the subjectivity and variability of the assessments, and that there is a clinical need for objective activity data. Key variables identified as most clinically useful were: step count, cadence and ability to change cadence, walking aid use, and ability to traverse different terrain types. Specific types of terrain of interest included flat ground, stairs, ramps, and uneven surfaces (e.g., grass, unstable gravel). It was also discovered that the issues mainly occur in decisions between K2 and K3 users.

Step count and cadence have been successfully assessed using a shank mounted inertia sensor for able-bodied and lower limb prosthetic users [7–11]. However, use of a walking aid in amputees has to-date not been classified, and terrain classification has only been attempted in healthy adults [12–15] and small groups of prosthetic users [16–19]. To classify terrain, wearable sensors have previously shown high accuracies when used on the lower limb prosthetic population [20]. Some previous studies have used sensors that would not be feasible for long-term real-world use [21–31]. A visual sensor, for example a depth camera or laser, would not be practical for real-world use as they need line of sight, thereby restricting what clothes a user could wear, but could be used to train a more practical sensor to reduce data processing. EMG sensors need to be attached to the user’s skin which is not practical for long-term real-world use, as this would require the prosthetic user to attach the electrodes. A few studies have shown that an IMU attached to a prosthesis can be used to classify certain terrain [32–34]. These studies only collected data from a small number of lower limb prosthetic users and did not incorporate real-world terrain. IMUs have also been shown to be able to classify terrain in larger healthy populations [35].

Machine learning offers the capability to classify human movement activities based on IMU data, but there is no conclusive agreement on the best algorithm to use or how to process the data, for example appropriate sampling rate or windowing method. Algorithms that have performed well in previous studies include Support Vector Machine (SVM) [16,19,36], Random Forest (RF) [37] and Long Short Term Memory neural networks (LSTM) [19,21,36]. Logistic Regression (LR) and K-Nearest Neighbours (KNN) have also previously been used and are considered simple models [38], hence could be used for benchmark comparisons. There is no conclusive evidence regarding which machine learning model would produce the highest terrain classification or walking aid recognition accuracies from wearable sensors, hence a comparison of these models is yet required for these purposes.

Similarly, there is currently no conclusion on window type and size: studies using time-based windows vary in window size from 4ms [39] to 8s [40], all producing similar accuracies. Some studies have produced high accuracies splitting the data into strides [12,41,42], but similar accuracies have also been reported in studies that have analysed individual datapoints [32,43]. Likewise, sampling rate which ranges from 20 Hz [36] to 500 Hz [28] in different studies produced similar results.

Sensor placement also varied in previous studies. To be minimally intrusive for a prosthetic user, a system would ideally be attached to the prosthesis and not the user’s body. Reported differences in knee and ankle kinematics across varying terrains indicate that an IMU positioned on the shank is likely to produce distinguishable signals [44]. Supporting this a single shank-mounted IMU can produce good terrain classification accuracies (0.90 F1-score) on able-bodied individuals [35], but this has not been investigated on lower limb prosthetic users. Good accuracies were also found with a trunk (0.84 F1-score) and thigh (0.89 F1-score) placed sensor with all sensors combined producing the highest accuracies (0.92 F1-score). This study used all attributes of the IMU in the models. Hence, comparison between prosthetic‑shank IMU with sensors on the thigh, contralateral shank and trunk is needed.

To assess accuracy of the classifiers, most studies used a cross-validation (CV) evaluation instead of a leave-one-out approach. This could have provided a less realistic real-world accuracy. The only previous study found that classified real-world terrain for lower limb prosthetic users using a wearable sensor (accelerometer) and assessed using a leave-one-out approach achieved a 56.68% mean accuracy, but this study had only recruited 4 prosthetic users and only classified between flat, stairs (ascending and descending) and slopes (ascending and descending) [45].

Walking aid recognition using wearable sensors has previously been investigated in able-bodied adults but only by using a wrist-worn accelerometer [46,47]. High accuracies have been achieved, but only in limited indoor environments so the performance on a variety of terrain has to-date not been investigated. Studies using gait-lab based 3D motion analysis have found differences in lower limb kinematics during strides with and without a walking aid on flat ground [48,49] and during stair climbing [50]. Considering that wearable sensors on the lower limbs are able to measure the kinematics, their use on the lower limbs should likewise be able to detect the differences between strides with and without a walking aid.

These studies show that inertial sensors and machine‑learning methods can classify human activities, but they use heterogeneous sensor placements, windowing strategies, feature sets and algorithms, often in small, laboratory‑based samples. For lower‑limb prosthesis users there is, to our knowledge, no validated, minimally obtrusive, single‑sensor system that can classify both terrain type and walking‑aid use under real‑world conditions. It is also unclear which combination of sensor placement, window definition, sampling density and IMU features offers the best trade‑off between classification performance and clinical feasibility.

The **aim** of this study was to investigate the use of a single prosthesis shank-mounted IMU for terrain classification and walking aid recognition in lower limb prosthetic users in the real-world and evaluate its potential clinical benefit. The **objectives** were:

- Compare machine learning algorithms for terrain classification and walking aid recognition. We hypothesised that algorithm performance would differ across models, but there is insufficient prior evidence to predict a single best‑performing algorithm; therefore, we treated this comparison as exploratory.
- To see how prosthetic shank IMU placement compares to other sensor locations (thigh, trunk and contralateral shank) and against using multiple sensors for terrain classification and walking aid recognition. We hypothesised that a single prosthetic‑shank IMU will achieve accuracies comparable to the other single sensors and a multi‑sensor configuration.
- Investigate how windowing method and sampling rates affect classification accuracies. We hypothesised that different windowing methods and sampling rates will lead to measurable differences in classification accuracies, but since existing evidence does not support a directional hypothesis, we treated this comparison as exploratory.
- Evaluate the impact of each feature of the IMU (accelerometer, gyroscope, magnetometer), and combinations of these, on classification accuracy. We hypothesised that including multiple features will improve or at least not degrade performance compared with single‑feature models, but there was no prior evidence to predict an optimal feature combination so we treated this as exploratory.
- Explore whether longitudinal real-world activity measures generated using the most promising data analysis approach are clinically meaningful to prescribing clinicians. Based on prior interviews with clinicians, we hypothesised that the real-world activity measures would be perceived as relevant to K‑level assessment.

## Methods

### Overall study design

To ensure clinical relevance of the sensor system, the associated research contained two phases: 1) development and training of a machine learning system to classify clinically relevant measures, and 2) clinical evaluation of the system by experts. The study had university and NHS IRAS ethical approval (4743 and 314743, respectively) and all participants provided informed consent.

### Phase 1: System development and data collection

#### Participants and data collection.

Participants were recruited through prosthetic organisations, charities, academic institutes and prosthetic clinics across the UK. Inclusion criteria for participants included ability to walk using their prosthesis unaided on the tested terrain and experience with using a walking aid. As the issues with K-level assessments predominantly occur with K2 and K3 users, we used the following exclusion criteria: not regularly participating in active sport with their prosthesis (football, running, etc.) and not primarily using a wheelchair; we note that this could limit the generalisability of the findings to K1 and K4 users. Twenty lower limb prosthetic users (mean±SD age: 64.6 ± 11.8 years, 2 female, 18 male; 11 transtibial, 8 transfemoral, 1 bilateral amputee) were recruited, participant details are shown in Table 1. The participant demographics did not perfectly match those of UK lower limb prosthetic users: 10% of participants were female versus approximately 30% nationally, and 85% were older than 54 years compared with around 70% in the UK population, however, the distribution of amputation levels was similar (transtibial 55% vs 54%, transfemoral 40% vs 37%, bilateral 5% vs 6%), reflecting representativeness of the data in that regard. For pragmatic reasons we did not sample to exactly mirror national demographics, which may limit generalisability of the findings, particularly with respect to sex‑specific and age‑related patterns of activity.

**Table 1:** Participant information and tests conducted for each participant. “wi”: data collected with walking aid, “wo”: data collected without walking aid.

Participant no.	Age	Amputation level	Sex	Flat	Grass	Stairs	Slope	Unstable	Uneven
**1**	85	TT	M	wi/wo	wo	wi/wo	wi/wo	wo	wi/wo
**2**	63	TT	M	wi/wo	wo	wi/wo	wi/wo	wo	wi/wo
3	74	TT	M	wi/wo			wi/wo		wi/wo
4	69	TT	M	wi/wo	wi/wo	wi/wo	wi/wo	wi/wo	wi/wo
5	56	TT	M	wi/wo		wi/wo	wi/wo		wi/wo
6	72	TT	M	wi/wo	wi/wo	wi/wo	wi/wo	wi/wo	wi/wo
7	69	TT	M	wi/wo	wi/wo	wi/wo	wi/wo	wi/wo	
8	33	TT	F		wi/wo	wi/wo	wi/wo		
9	62	TT	F		wo	wo	wo		
10	61	TT	M		wo	wo	wo		
11	65	TT	M		wo	wo	wo		wo
**12**	69	TF	M	wi/wo		wi/wo	wi/wo	wi/wo	wi/wo
**13**	80	TF	M	wo	wo	wo	wo	wo	wo
**14**	53	TF	M	wi/wo	wi/wo	wi/wo	wi/wo	wi/wo	wi/wo
**15**	59	TF	M	wi/wo	wi/wo	wi/wo	wi/wo	wi/wo	wi/wo
**16**	64	TF	M	wi/wo	wi/wo		wi/wo	wi/wo	wi/wo
**17**	44	TF	M		wo	wo	wo		wo
**18**	67	TF	M	wi	wi	wi	wi		
**19**	80	TF	M		wi/wo	wo		wi/wo	
20	66	TT/TF	M	wi/wo		wi/wo	wi/wo	wi/wo	wi/wo

Each participant wore four Xsens Awinda IMUs (100 Hz sampling rate) placed on the anterior prosthetic shank, thigh, the contralateral shank (approximately at the midpoint of each limb segment, see Fig 1). A further IMU was placed on the trunk at the approximate location of the L4 vertebra as shown in Fig 1. Prosthetic shank placement varied depending on the participant’s prosthesis; however, the IMU was placed on the prosthesis pylon to ensure consistent orientation. For each IMU, raw tri‑axial acceleration, free acceleration, angular velocity and magnetic field signals were used. Cadence signals were also calculated from the step count. Free accelerations are the acceleration signals with the effect of gravity removed. The resultant acceleration and free acceleration signals were also calculated. Magnetic field strength was included because it provides complementary information on sensor heading and orientation relative to the environment, which may differ across variable terrain (e.g., slopes vs level ground). It is not known which signals are the most important for terrain classification and walking aid recognition. Sensors were mounted so that the local X, Y and Z axes were approximately aligned with the segment’s vertical, medio-lateral and anterior–posterior directions, respectively. The Xsens Awinda IMUs incorporate factory calibration for accelerometer, gyroscope, and magnetometer sensors; no additional user‑level calibration procedure was applied before each session.

**Fig 1:**
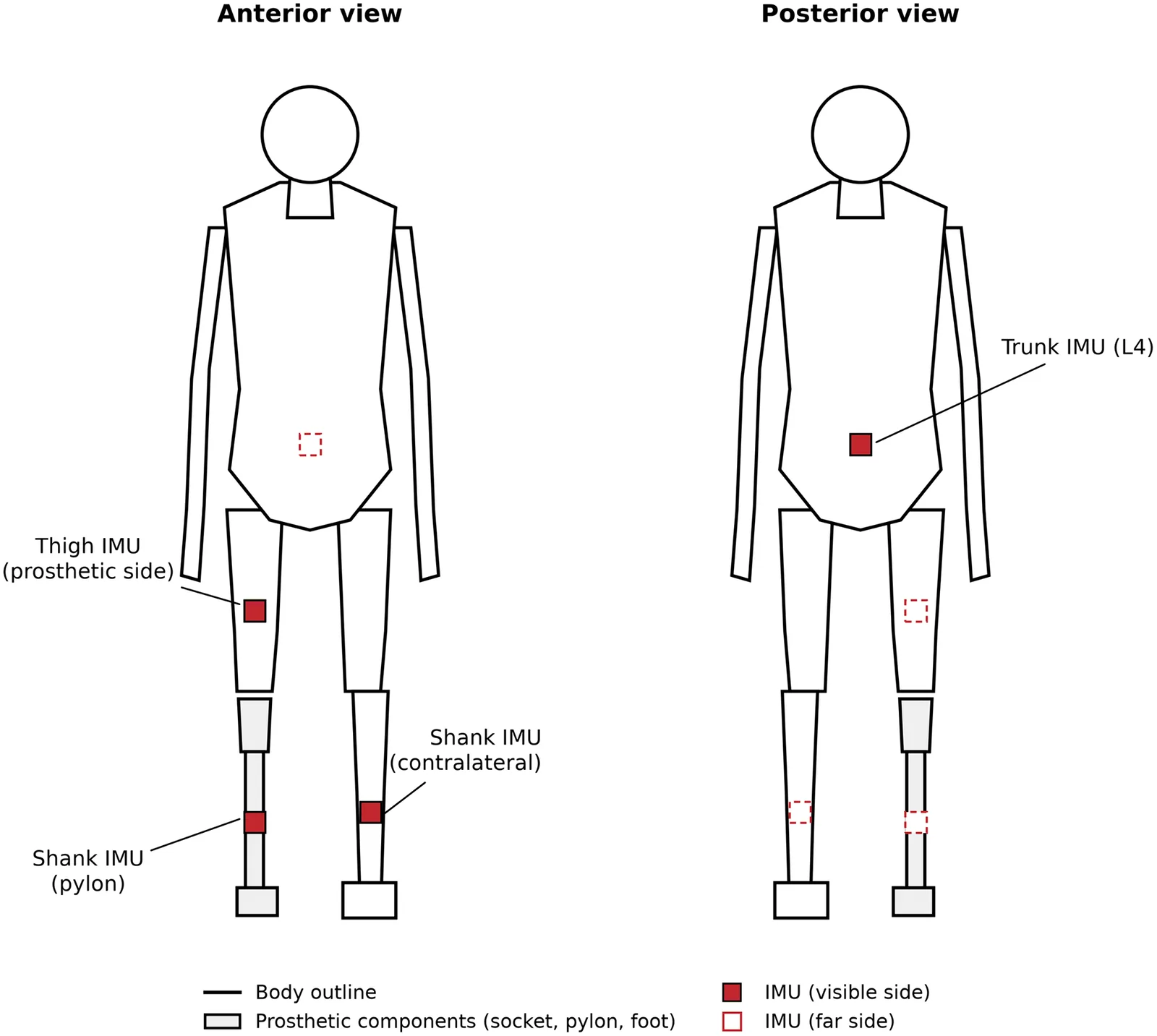
IMU placement.

Participants walked over designated sections of various types of terrain (flat ground, stairs, ramp, grass, uneven ground, unstable gravel) with and without a walking aid in separate trials. The order of the terrain and walking aid condition was randomised for each participant. The walking aid used in the study was a walking stick. The only unstable terrain used in the data collection was gravel, assessed by the researcher to be a loose, small‑stone surface deep enough that the stones moved underfoot and visibly deformed with each step. A ramp was classified as a slope steeper than 1:20 [51]. Uneven ground was hard terrain with variable footing heights and/or angles that the researcher conducting the study deemed to be uneven, for example cobblestones, this may reduce reproducibility and this subjectivity is revisited in the Discussion as a potential contributor to misclassification. Not all the participants were assessed on every terrain, due to terrain conditions. Four participants did not feel comfortable using the walking aid, and one participant only felt comfortable conducting the data collection with a walking aid. Data collection conditions for each participant are displayed in Table 1. Depending on the length of the terrain available and the participant’s comfort on the terrain, the number of separate trials conducted on each terrain varied between 2 and 8. Fig 2 shows representative terrain used in the data collection. The total number of strides recorded on each terrain with and without a walking aid is shown in Table 2. The number of recorded strides per terrain class was imbalanced, reflecting the practical difficulty of collecting data on different types of terrain. Class imbalance may have contributed to poorer performance for under-represented or more heterogeneous classes, but the risk was considered limited in this dataset due to the marked biomechanical differences in traversing the different types of terrain. In the discussion individual class imbalances are discussed. Participants walked at their self-selected natural walking speed on each terrain, which increases ecological validity of the dataset. The total length of a single data collection session was less than 2 hours and participants were encouraged to rest between trials when needed. The data were filtered using a 5 Hz low-pass Butterworth filter, applied to all IMU signals. The cut‑off was selected based on a frequency analysis of resultant acceleration from a representative subset of strides across terrains and walking aid conditions, which showed the dominant gait‑related power to be below 4 Hz. A combination of per‑person normalised and non-normalised data were used in the models to stabilise training while preserving absolute between‑subject differences. Normalisation is known to improve optimisation and reduce sensitivity to scale, but purely person‑specific normalisation can attenuate clinically relevant between‑subject variation; including both representations was therefore chosen as a pragmatic compromise in this feasibility study [52,53]. To our knowledge, combining per‑person normalised and raw IMU channels within the same feature set has not been reported previously, but in preliminary analysis [6] this combination produced the most favourable balance of cross‑validation and leave‑some‑out accuracies, so it was adopted for the present models.

**Fig 2:**
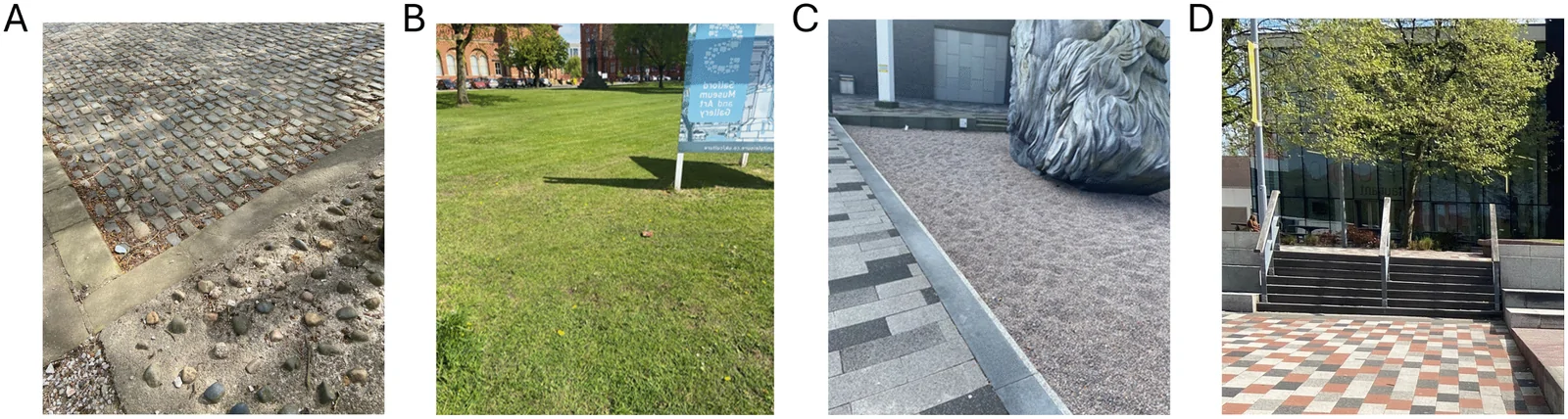
Representative terrain used in the data collection. A) Uneven ground (cobblestones), B) Grass, C) Unstable gravel, D) Stairs.

**Table 2:** Number of strides recorded on each terrain with and without a walking aid.

	Without walking aid	With walking aid	Total
Flat	1603	1348	2951
Grass	671	340	1011
Stairs ascending	412	288	700
Stairs descending	423	287	710
Ramp ascending	646	293	939
Ramp descending	711	331	1042
Uneven ground	858	560	1418
Unstable gravel	603	343	946
Total	5927	3790	9717

#### Model development.

A comparative analysis was performed to determine the optimal machine learning model for terrain classification and walking aid recognition. The algorithms tested were SVM, KNN, RF, and LSTM. For walking aid recognition, a LR classifier was also used as a baseline. The following model parameters were investigated to optimise performance:

- Sensor Placement: Data from IMUs placed on the prosthetic shank, prosthetic leg thigh, contralateral shank and trunk were compared as well as data from all IMUs combined were also evaluated.
- Windowing: Multiple time-based non-overlapping windows (0.1s to 2s) were evaluated alongside windows comprising a full stride versus individual datapoints. The individual datapoint windows were not tested using cross-validation as datapoints from the same stride would otherwise be split across train and validation sets, leading to severe information leakage and artificially high accuracies. The sampling rate or number of samples per stride was also examined.
- Feature Selection: Various combinations of IMU features (acceleration, angular velocity, magnetic field strength, free acceleration, cadence) were evaluated.

To label and segment the data into stride-based windows, we required not only accurate stride detection but also sufficient temporal precision to identify a consistent point within each stride, ensuring that each window contained a complete stride. Preceding analysis [6] established that the rate of turn along the medio-lateral direction of the prosthetic shank produces an accuracy of 96.15% and a precision of 97.97%, and was hence used to detect strides in the data processing. Strides were detected during the mid-swing phase of gait, and each stride was labelled according to the terrain under the prosthetic foot at initial contact. Steps that transition between different types of terrain were not separately identified. Step count was determined using the same method as stride detection with an accuracy of 96.15% [6]. The time taken for each stride was used to calculate the cadence for individual strides, from which mean cadence and cadence variability were derived.

Participants included transtibial, transfemoral and one bilateral amputee, and all models were trained on the combined dataset without stratification by amputation level. Analyses were carried out that did not show significant differences in classification accuracy between transtibial and transfemoral subgroups [6], however, the present study cannot exclude that performance varies by amputation level and this remains a limitation.

For each algorithm a Bayesian hyperparameter optimiser was run to mitigate overfitting. The optimisation was run on a representative subset of the data to obtain reasonable default settings. The optimiser evaluated a loss function on held‑out data, seeking the hyperparameter combination that minimised this loss. This initial optimisation was performed once per algorithm using 100 Hz data with 0.2 s windows and stride‑based windows (100 datapoints per stride). The optimal values were very similar across window types, and where small differences (<5%) occurred the mean of these values were used.

Analysis was conducted sequentially so the condition or processing method that produced the highest accuracy with consideration also given to reducing the computational need from a step was then used for the sequential steps in the analysis. The initial condition used for the sensor placement analysis was 100 Hz data, split into windows that contained a whole stride split into 100 datapoints.

#### Performance evaluation.

Overall percentage accuracy was used as the primary optimisation metric because the target application is multi‑class terrain and walking aid recognition, where correct class assignment across all categories is clinically most relevant and directly comparable with prior studies [17,18,20,45]. Accuracy was calculated for each validation test set as the proportion of held-out stride windows correctly classified. Per-class performance is reported via confusion matrix for the final model, with precision, sensitivity and specificity reported. For each class, sensitivity was calculated as TP/(TP+FN), precision as TP/(TP+FP), and specificity as TN/(TN+FP), where TP, TN, FP and FN denote true positives, true negatives, false positives and false negatives, respectively; metrics were calculated using a one-versus-rest approach. Model accuracy was determined using both 5-fold CV and a more robust 5-group leave-some-out approach (LSO), which is similar to a leave-one-out approach, but participants are quasi-randomly assigned to groups to ensure a mix of prosthetic types and reduce computation need. The mean accuracy across the five test folds/groups are reported for both LSO and CV, with standard deviation (SD) calculated across fold/group accuracies and 95% confidence intervals (CI) calculated using the Student’s t distribution ($\pm t_{\text{0}.\text{9}\text{7}\text{5},\text{4}}\times SD/\sqrt{\text{5}}$). Before comparing model accuracies, normality was assessed for each set of values using the Shapiro–Wilk test. When all models for a given comparison (e.g., windowing method, IMU position, feature set) satisfied the normality assumption (p ≥ 0.05), a one‑way ANOVA (with F-value (F), degrees of freedom and p-value (p) reported) was used with Tukey’s HSD post‑hoc tests. Kruskal-Wallis (with Chi-square (X^2^), degrees of freedom and p values (p) reported) with Dunn’s post-hoc test was used for non-parametric data. Significance was accepted as p values lower than 0.05. For significant ANOVA effects we report partial eta‑squared as an effect size; for key pairwise post‑hoc contrasts we additionally report Cohen’s d.

### Phase 2: Clinical evaluation of the system by experts

Real-world data were collected unsupervised over two weeks using a prosthesis shank-mounted IMU on 3 lower limb prosthetic users that had already participated in Phase 1. These participants also had their K-levels assessed by a clinician who had 8 years’ experience in conducting K-level assessments using standard clinical practice. The IMU data were processed using the optimal machine learning model developed in Phase 1. A report that documented daily measures for number of steps, number of steps on each type of terrain, mean cadence, steps above 20% mean cadence and number of steps taken with a walking aid and a separate summary of an average for each measure over the recoding period, along with a summary of the participants K-level assessment, was presented to 4 clinical experts (mean experience 21.5 years, 3 had participated in the preceding interviews that had provided knowledge in the clinical need [6]. Semi-structured interviews were then conducted with the clinical experts to obtain their evaluation of the system and the clinical relevance of the data it produced. Interviews were analysed using thematic analysis based on a framework approach described by Braun and Clarke [54]. Details of the interviews and thematic analysis can be found in supplementary file S1 File.

## Results

The mean accuracies for all models tested for terrain classification and walking aid recognition are presented in Table 3, but the main findings are summarised in the text that follows.

**Table 3:** Mean percentage accuracy for each model analysed (standard deviation shown in brackets), for terrain classification and walking aid recognition.

Test	Terrain classification								Walking aid recognition									
	KNN		RF		SVM		LSTM		KNN		RF		SVM		LSTM		LR	
	LSO	CV	LSO	CV	LSO	CV	LSO	CV	LSO	CV	LSO	CV	LSO	CV	LSO	CV	LSO	CV
IMU position																		
Prosthetic Shank	44.19 (3.92)	84.30 (0.63)	52.09 (3.48)	81.66 (0.72)	52.87 (6.40)	84.29 (0.44)	47.53 (3.75)	81.99 (0.40)	55.08 (3.84)	74.31 (0.80)	61.45 (8.33)	79.39 (0.82)	64.39 (7.15)	82.21 (0.49)	54.59 (7.12)	69.29 (1.26)	53.70 (6.66)	65.15 (0.43)
Contralateral Shank	43.52 (2.83)	83.32 (0.73)	53.56 (4.38)	80.60 (1.27)	52.41 (7.42)	82.11 (1.44)	46.62 (7.63)	82.20 (0.62)	52.44 (1.73)	75.22 (0.57)	61.43 (9.78)	79.11 (0.45)	62.58 (7.94)	83.97 (0.69)	56.55 (6.36)	70.79 (0.92)	55.35 (5.00)	65.76 (0.96)
Thigh	37.53 (2.64)	84.72 (0.90)	44.36 (6.38)	82.11 (0.82)	43.95 (6.61)	83.15 (0.88)	39.64 (4.65)	83.21 (0.90)	54.83 (2.65)	77.24 (0.83)	65.50 (12.71)	80.29 (1.08)	67.95 (13.53)	84.84 (0.68)	59.84 (6.56)	68.91 (0.73)	55.65 (10.90)	58.01 (0.76)
Trunk	43.51 (2.11)	81.67 (0.91)	54.30 (6.41)	79.90 (0.82)	53.22 (4.58)	81.36 (0.84)	50.31 (5.59)	81.48 (0.77)	53.48 (3.01)	76.63 (0.89)	60.68 (9.45)	79.41 (0.73)	60.61 (7.01)	83.45 (0.50)	56.49 (6.30)	71.43 (1.29)	48.43 (5.47)	65.24 (0.46)
All	44.73 (4.39)	82.68 (0.69)	52.00 (3.77)	84.92 (0.72)	49.08 (3.25)	87.32 (0.76)	50.06 (5.31)	81.61 (0.85)	51.40 (3.62)	78.21 (1.23)	66.50 (14.31)	81.71 (0.90)	67.45 (13.77)	75.04 (0.34)	64.45 (10.71)	65.61 (2.48)	48.99 (4.78)	53.19 (1.60)
Windowing method																		
0.1s	43.97 (3.92)	83.40 (0.18)	43.39 (2.00)	79.70 (0.13)	37.46 (2.97)	48.58 (0.47)	48.05 (3.73)	76.75 (0.44)	58.12 (6.99)	77.96 (0.26)	61.64 (8.29)	81.88 (0.31)	58.64 (8.60)	79.53 (0.16)	63.46 (8.06)	65.64 (0.09)	61.55 (8.63)	62.18 (1.24)
0.2s	39.99 (0.72)	77.68 (0.37)	43.04 (1.84)	74.32 (0.45)	41.56 (5.01)	51.50 (0.30)	44.31 (4.43)	54.94 (0.74)	57.88 (6.11)	73.55 (0.26)	61.81 (8.43)	76.96 (0.39)	59.01 (6.77)	77.19 (0.39)	54.85 (11.10)	55.69 (3.50)	61.68 (6.83)	61.05 (0.07)
0.5s	39.99 (0.72)	68.26 (0.64)	43.11 (2.01)	69.53 (0.60)	41.56 (5.00)	61.59 (0.26)	44.46 (2.59)	69.32 (0.41)	56.08 (6.93)	69.20 (0.59)	62.19 (8.99)	72.90 (0.54)	60.63 (7.98)	72.01 (0.78)	48.13 (4.79)	56.23 (2.34)	59.14 (7.33)	62.74 (1.09)
1s	36.49 (1.50)	63.01 (0.58)	43.01 (2.27)	62.79 (0.84)	43.24 (5.53)	64.54 (0.56)	39.40 (3.74)	67.15 (0.81)	53.89 (3.12)	77.78 (0.66)	61.55 (8.19)	74.96 (0.27)	62.68 (7.71)	78.61 (0.70)	50.91 (3.96)	72.35 (0.81)	61.79 (8.09)	66.24 (0.83)
1.5s	39.72 (1.37)	60.28 (1.53)	42.88 (1.53)	60.85 (0.63)	39.09 (5.91)	63.98 (1.38)	38.60 (3.10)	67.00 (0.77)	52.77 (1.94)	70.56 (1.08)	61.15 (6.46)	66.06 (1.22)	61.96 (7.86)	68.97 (0.73)	49.93 (1.70)	50.65 (1.18)	59.52 (6.76)	60.20 (2.75)
2s	36.35 (1.10)	56.70 (0.72)	42.11 (1.53)	56.08 (0.67)	37.53 (4.39)	57.72 (0.63)	37.03 (2.09)	61.93 (0.77)	51.99 (1.22)	64.40 (1.01)	61.64 (8.29)	68.81 (0.93)	61.46 (8.28)	65.87 (1.15)	50.73 (1.60)	50.32 (0.82)	57.48 (5.26)	61.09 (1.73)
Stride	44.19 (3.92)	84.30 (0.63)	52.09 (3.48)	81.66 (0.72)	52.87 (6.40)	84.29 (0.44)	47.53 (3.75)	81.99 (0.40)	55.08 (3.84)	74.31 (0.80)	61.81 (8.43)	79.39 (0.82)	64.39 (7.15)	82.21 (0.49)	53.70 (6.66)	65.15 (0.43)	54.59 (7.12)	69.29 (1.26)
Individual	44.42 (6.16)		46.52 (3.54)		32.72 (3.53)		48.23 (7.07)		59.52 (6.67)		62.19 (8.99)		62.65 (9.22)		54.39 (10.09)		60.43 (8.29)	
Number of datapoints																		
10	45.45 (3.28)	85.27 (0.24)	54.40 (4.01)	82.59 (1.08)	52.10 (4.23)	77.31 (0.37)	45.58 (4.47)	80.78 (0.82)	55.97 (5.45)	74.85 (0.71)	60.07 (9.91)	78.71 (0.92)	59.61 (6.52)	83.10 (0.42)	53.61 (7.63)	70.86 (1.34)	57.02 (5.41)	66.66 (2.31)
20	44.55 (3.57)	85.02 (0.39)	53.13 (4.07)	82.32 (0.67)	51.78 (4.90)	79.95 (0.90)	47.35 (4.33)	81.65 (0.95)	55.17 (4.43)	74.79 (0.37)	62.07 (8.42)	79.60 (0.53)	60.22 (6.91)	83.32 (0.80)	54.64 (10.6)	73.25 (0.55)	55.96 (5.20)	67.75 (2.11)
50	44.31 (3.71)	84.34 (0.53)	53.25 (4.81)	81.62 (0.32)	52.10 (6.29)	82.10 (0.52)	47.39 (2.06)	82.23 (0.80)	54.69 (4.23)	74.60 (0.81)	61.99 (8.61)	79.25 (0.37)	62.74 (7.81)	83.28 (0.78)	50.90 (9.32)	71.78 (0.52)	55.57 (9.23)	67.39 (1.40)
100	44.19 (3.92)	84.30 (0.63)	52.09 (3.48)	81.66 (0.72)	52.87 (6.40)	84.29 (0.44)	47.53 (3.75)	81.99 (0.40)	55.08 (3.84)	74.31 (0.80)	61.45 (8.33)	79.39 (0.82)	64.39 (7.15)	82.21 (0.49)	53.70 (6.66)	65.15 (0.43)	54.59 (7.12)	69.29 (1.26)
Variables																		
Accelerations	36.80 (2.16)	76.46 (0.36)	44.12 (4.04)	76.26 (0.34)	42.14 (2.53)	58.96 (0.69)	40.36 (2.43)	71.60 (1.28)	54.81 (3.14)	74.96 (1.18)	65.04 (6.42)	77.84 (0.89)	59.76 (11.12)	78.94 (0.45)	59.58 (7.71)	72.36 (0.99)	56.56 (8.19)	66.94 (1.22)
Angular Velocity	29.47 (4.39)	73.66 (1.84)	36.32 (3.03)	74.07 (0.50)	29.03 (2.65)	48.26 (0.53)	32.86 (2.60)	62.75 (0.56)	57.40 (3.66)	75.48 (0.69)	55.68 (10.80)	76.80 (0.55)	58.39 (6.97)	76.54 (0.81)	56.98 (8.90)	68.23 (1.01)	55.47 (10.92)	63.34 (1.52)
Magnetic Field Strength	28.57 (3.14)	80.77 (0.43)	41.48 (4.58)	79.47 (0.65)	35.80 (2.69)	47.98 (0.58)	34.00 (6.30)	66.74 (1.25)	54.65 (4.74)	70.00 (1.27)	60.66 (7.52)	72.90 (0.42)	55.32 (6.29)	68.59 (0.99)	57.63 (6.03)	67.02 (1.47)	55.03 (11.07)	62.32 (1.34)
Free Accelerations	40.91 (2.29)	80.04 (0.63)	50.03 (4.04)	77.52 (0.25)	48.60 (3.73)	57.54 (1.09)	46.22 (3.51)	74.16 (0.75)	55.25 (3.37)	73.56 (0.66)	61.33 (6.32)	73.92 (0.44)	59.24 (6.39)	73.87 (0.42)	56.47 (8.80)	66.99 (0.69)	57.02 (5.02)	64.27 (1.95)
AVM	41.82 (4.18)	84.85 (0.85)	49.49 (4.07)	82.68 (1.13)	49.39 (4.12)	70.08 (1.04)	41.15 (5.87)	79.49 (1.73)	55.27 (4.38)	74.31 (0.41)	60.67 (10.44)	79.00 (0.55)	59.56 (9.76)	82.73 (0.68)	56.10 (9.59)	74.29 (1.01)	62.00 (5.70)	65.54 (0.94)
AVMF	44.87 (3.82)	85.67 (1.04)	54.18 (3.35)	83.18 (0.85)	52.28 (3.94)	74.44 (0.88)	43.32 (2.94)	80.18 (1.60)	55.63 (3.90)	74.30 (0.37)	60.58 (9.14)	78.16 (0.46)	59.63 (8.11)	82.01 (0.73)	54.75 (9.70)	74.44 (0.75)	55.58 (3.22)	64.14 (1.16)
AVMFC	45.46 (2.82)	85.61 (0.63)	54.36 (3.79)	82.83 (0.25)	52.83 (3.98)	75.17 (1.09)	46.94 (4.09)	80.59 (0.75)	55.51 (4.57)	75.49 (0.55)	60.97 (8.94)	78.54 (0.79)	59.55 (8.06)	82.90 (0.72)	54.70 (9.70)	74.46 (0.39)	59.92 (7.70)	63.36 (2.17)
All	45.45 (3.28)	85.27 (0.24)	54.40 (4.01)	82.59 (1.08)	52.10 (4.23)	77.31 (0.37)	45.58 (4.47)	80.78 (0.82)	55.97 (5.45)	74.85 (0.71)	60.07 (9.91)	78.71 (0.92)	59.61 (6.52)	83.10 (0.42)	53.61 (7.63)	70.86 (1.34)	57.02 (5.41)	66.66 (2.31)

KNN = K-Nearest Neighbours, RF = Random Forest, SVM = Support Vector Machine, LSTM = Long Short-Term Memory neural network, LR = Logistic Regression, AVM = Acceleration Angular Velocity + Magnetic Field Strength, AVMF = Acceleration + Angular Velocity + Magnetic Field Strength + Free acceleration, AVMFC = Acceleration + Angular Velocity + Magnetic Field Strength + Free acceleration + Cadence.

### Terrain classification

Comparing IMU location it was found that there were no significant differences in classification accuracies between IMU positions for LSO results. For CV results IMU position had a significant effect for all the algorithms except for LSTM (KNN X^2^(4)=17.60 p = 0.002, RF X^2^(4)=17.25 p = 0.002 and SVM X^2^(4)=19.62 p < 0.001). The ‘all- combined’ produced significantly higher accuracies than using any IMU by itself; for RF models all-combined compared to prosthesis shank IMU the mean difference was 3.26% (*p* < 0.001, Cohen’s $d_{z}=\text{2}.\text{7}\text{0}$). A minimally obtrusive system would be prosthesis shank-mounted, and using this single IMU did not show significantly lower accuracies than ‘all-combined’ IMU or any other single IMU for any LSO or the KNN, SVM and LSTM CV models. Only data from the single prosthesis shank IMU were hence used for the rest of the analysis.

Windowing method had a significant effect on terrain‑classification accuracy for all four algorithms for LSO (KNN X^2^(7)=25.34 p < 0.001, RF X^2^(7)=16.32 p < 0.001, SVM X^2^(7)=19.08 p < 0.001 and LSTM X^2^(7)=20.39 p < 0.001) and CV tests (KNN X^2^(6)=33.33 p < 0.001, RF X^2^(6)=33.24 p < 0.001, SVM X^2^(6)=32.69 p < 0.001 and LSTM X^2^(6)=32.74 p < 0.001). The stride-based windows produced the highest accuracies for RF and SVM LSO tests (RF 52.09%, SVM 52.89%) and CV tests (RF 81.66%, SVM 84.29%). KNN and LSTM showed similar trends. On this basis, stride‑based windows were used in subsequent analyses. The number of datapoints per window showed a significant effect for only KNN and SVM CV results (KNN X^2^(3)=8.47 p = 0.0372, SVM X^2^(3)=17.86 p < 0.001). Post-hoc tests indicated that reducing the windows from 100 datapoints to 10 significantly reduced the SVM CV accuracy (mean difference 6.98%, *p* < 0.001, Cohen’s $d_{z}=\text{8}.\text{1}\text{3}$), whereas RF and KNN accuracies were less sensitive to sampling density. The 10 datapoint model was hence used for the rest of the analysis.

The features used in a model had significant effects for both LSO (KNN X^2^(8)=29.31 p < 0.001, RF X^2^(8)=27.01 p < 0.001, SVM X^2^(8)=29.82 p < 0.001 and LSTM X^2^(8)=21.05 p = 0.004) and CV (KNN X^2^(8)=34.29 p < 0.001, RF X^2^(8)=33.59 p < 0.001, SVM X^2^(8)=37.57 p < 0.001 and LSTM X^2^(8)=34.24 p < 0.001). Combining features produced higher LSO and CV accuracies compared to single feature models. Combining accelerations, angular velocity, magnetic field strength, free accelerations and cadence (AVMFC) data together produced the highest mean accuracy for KNN, SVM and LSTM LSO tests and produced the second highest for all CV tests. Combining accelerations, angular velocity, magnetic field strength and free accelerations did produce higher CV accuracies for KNN and RF but lower LSO accuracies for all tests, but none of these differences were significant. Hence AVMFC was chosen to be used in the successive analysis.

Based on the LSO accuracy, the RF model was selected as the final classifier for terrain classification. While the KNN model produced a slightly higher CV accuracy, the RF model achieved higher LSO accuracy. Because LSO withheld participant groups during training, it was considered a more conservative estimate of performance in unseen users than the CV estimate. An additional Bayesian hyperparameter optimisation was run on the final RF model (prosthetic shank IMU, 10 data point stride-based windows, AVMFC features) and the re-optimised model achieved a mean LSO accuracy across held-out participant groups of **56.89% (SD 5.5%; 95% CI 50.1–63.7%)** and a mean CV accuracy of **83.64% (SD 0.8%; 95% CI 82.6–84.7%)**.

To investigate participant variability leave-one-participant-out (LOPO) analysis was run using the final RF model, Table 4. Mean accuracies between amputation levels were similar (TT 51.8 ± 14.6%, TF 50.4 ± 20.0%), and Welch’s t‑test showed no significant difference between groups (p = 0.86), suggesting that amputation level did not influence classification accuracy.

**Table 4:** LOPO classification accuracies.

Participant	Amputation level	Terrain classification accuracy	Percentage of steps with a walking aid	Walking aid recognition accuracy
P1	TT	71.71%	19.02%	54.34%
P2	TT	64.19%	25.22%	62.09%
P3	TT	40.34%	44.02%	22.32%
P4	TT	54.24%	49.64%	61.86%
P5	TT	28.77%	22.43%	89.62%
P6	TT	32.54%	50.93%	49.04%
P7	TT	67.55%	50.36%	53.98%
P8	TT	42.83%	60.17%	47.24%
P9	TT	44.87%	0.00%	59.40%
P10	TT	65.56%	0.00%	66.70%
P11	TT	63.31%	0.00%	82.32%
P12	TF	71.47%	50.77%	60.78%
P13	TF	33.81%	0.00%	51.80%
P14	TF	40.54%	52.06%	18.92%
P15	TF	24.48%	47.06%	41.15%
P16	TF	22.40%	43.10%	34.90%
P17	TF	62.61%	0.00%	58.32%
P18	TF	59.61%	100.00%	96.31%
P19	TF	70.18%	35.42%	56.96%
P20	TT	45.30%	54.01%	29.91%
	TF	68.16%	55.23%	59.34%

### Walking aid recognition

IMU location did not have a significant effect on LSO accuracies but did significantly affect CV accuracies (KNN X^2^(4)=18.35 *p* = 0.0011, RF X^2^(4)=12.87 *p* = 0.012, SVM X^2^(4)=20.53 *p* < 0.0001, LR X^2^(4)=15.49 *p* = 0.0038, LSTM X^2^(4)=19.14 *p* < 0.0001). The trunk IMU using a SVM model produced the highest LSO and CV accuracy, but there were no significant differences between IMU position for any of the LSO models. A minimally obtrusive system would be prosthesis shank-mounted. Using this single IMU was only significantly worse than the trunk IMU for the SVM and KNN models, but the difference was small (SVM 2.63% *p* = 0.034 Cohen’s $d_{z}=\text{2}.\text{5}\text{2}$, KNN 2.93% *p* = 0.026 Cohen’s $d_{z}=\text{5}.\text{0}\text{0}$). Data from the single prosthetic shank IMU only was therefore used for the rest of the analysis.

Windowing method had no significant effect on LSO but did significantly affect CV accuracies (KNN X^2^(6)=32.01 *p* < 0.001, RF X^2^(6)=33.16 *p* < 0.001, SVM X^2^(6)=32.98 *p* < 0.001, LR X^2^(6)=31.82 *p* < 0.001, LSTM X^2^(6)=25.13 *p* < 0.001). Stride-based windows produced the highest accuracies for the LSO and CV tests using a SVM model, and no stride-based window model produced accuracies that were significantly lower than any other windowing method. Stride-based windows were hence used for the sequential analysis. Reducing the windows to 10 datapoints did not significantly reduce the LSO or CV accuracies for any model. The 10 datapoint model produced the highest KNN and LSTM LSO accuracies and was therefore used for the rest of the analysis.

The features included in the models had no significant effect on the accuracies of any LSO model but did show a significant effect on CV accuracies (KNN X^2^(8)=35.51 *p* < 0.001, RF X^2^(8)=38.09 *p* < 0.001, SVM X^2^(8)=42.18 *p* < 0.001, LR X^2^(8)=18.50 *p* = 0.018, LSTM X^2^(8)=32.48 *p* < 0.001). Combining features generally produced higher CV accuracies compared to single feature models. Combining accelerations, gyroscope, magnetometer, free accelerations and cadence data together produced consistently high LSO and CV accuracies for all models and did not produce significantly lower accuracies than any other feature combination for any LSO or CV model, so only these features were used in successive analysis.

The SVM model provided the best performance for walking aid recognition. It had the highest CV accuracy and showed no significant difference from the other models in LSO accuracy, making it the most reliable choice. The final SVM model (prosthetic shank IMU, 10 data point stride-based windows, AVMFC features) was re-optimised and achieved a mean LSO accuracy across held-out participant groups of **61.56% (SD 16.1%; 95% CI 45.4–77.7%)** and a mean CV accuracy of **82.90% (SD 2.1%; 95% CI 80.8–85.0%)**.

LOPO walking aid recognition accuracies showed a large inter participant variability, Table 4, but amputation level did not affect model accuracies (TT 56.6 ± 19.0%, TF 53.2 ± 21.4%; Welch’s t‑test p = 0.71). The proportion of steps performed with a walking aid was also not significantly associated with walking aid recognition accuracy (Pearson r=−0.10, p = 0.65; Spearman p=−0.29, p = 0.20), suggesting that uneven exposure to aided steps did not systematically bias performance.

### Phase 2: Clinical evaluation

Detailed results from the thematic analysis can be found in supplementary file S1 File. All the interviewees thought the system was clinically relevant and when comparing the system data to the clinical assessment summaries they thought that the sensor data could have changed the K-level for some of the participants that they otherwise would not have assigned to them. They all thought the data would be very useful to have to add to their clinical assessment, to justify decisions, prompt in-depth questions on patients’ lives, and enable difficult conversations to be had objectively. The use of the data to prompt questions was a positive that the interviewees were very enthusiastic about, as it would allow them to get more information about their patients and, therefore, a greater understanding of their lives, which could help them ensure that their patients are getting the right prescription and support.

The ability to distinguish between different types of terrain was particularly appreciated, as this provided a new level of detail for assessing a patient's activity. The primary criticism of the system was distinguishing between different grass, uneven and unstable terrain. Some of the clinical experts suggested that combining these types of terrains would make the data easier to understand. Additional measures of loading through the prosthesis and wear-time were suggested to add to the clinical relevance of the system.

In summary, the clinical evaluation of the developed system was highly positive. The clinicians perceived the system as a useful tool for quantifying K-level assessments. The data provided by the system was deemed to be highly valuable, as it provided objective, real-world data that was previously unobtainable.

## Discussion

This study has suggested that a single prosthesis-mounted IMU can be used to measure clinically relevant variables, specifically step-count, cadence and terrain classification, which could improve lower limb prosthesis prescriptions. The findings confirm a strong desire from clinicians to obtain this objective data to support and enhance K-level decisions.

The final RF model produced a LSO accuracy of 56.89%, Fig 3, but the model overpredicted for flat terrain (50.8% precision, 67.2% specificity and 83.4% sensitivity) but the precision of steps predicted as stair or ramp ascending/descending is much higher (stair ascending 81.8%, stair descending 77.5%, ramp ascending 73.5%, ramp descending 68.1%). Low precision values were observed in grass and uneven terrain classifications which were commonly misclassified as each other or flat terrain. The final RF CV model, Fig 4, also overclassified for flat terrain (73.8% precision, 86.5% specificity and 92.4% sensitivity) but produced high precision for all other terrain classifications (grass 87.8%, stair ascending 90.1%, stair descending 91.2%, ramp ascending 93.8%, ramp descending 89.7%, uneven 86.4%, unstable 91.1%). LOPO accuracies ranged from 22.4% to 71.7% indicating considerable inter‑individual variability. This indicates that while the classifier generalises reasonably, performance for some users is lower and may require user‑specific calibration or a larger more diverse training dataset. The clinical experts in Phase 2 suggested combining grass, uneven and unstable terrain categories into one uneven terrain category. In response to their comment, a RF model with these terrain categories was created for supplementary data analysis which then produced an accuracy increase to 65.70% (SD 6.6%; 95% CI 47.3–84.1%) for LSO and to 85.65% (SD 0.79%; 95% CI 83.4–87.9%) for CV. This increase in accuracy is purely down to the high accuracy in the combined uneven class (LSO precision 60.4%, sensitivity 77.9% and specificity 75.7%) with the accuracies from the other classes essentially unchanged, and per‑class sensitivity ranged from approximately 44–78%.

**Fig 3:**
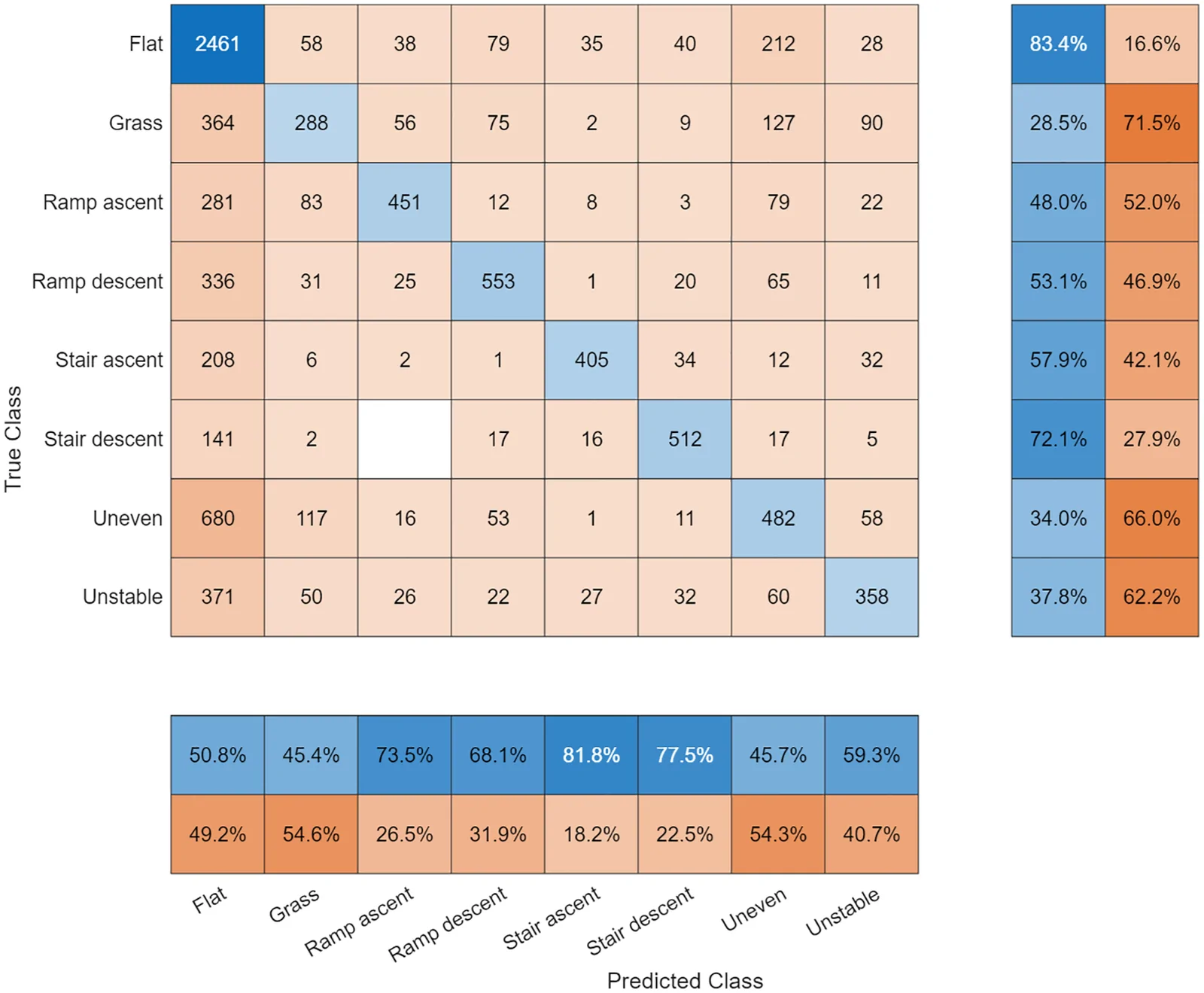
Confusion matrix for terrain classification RF leave-some-out.

**Fig 4:**
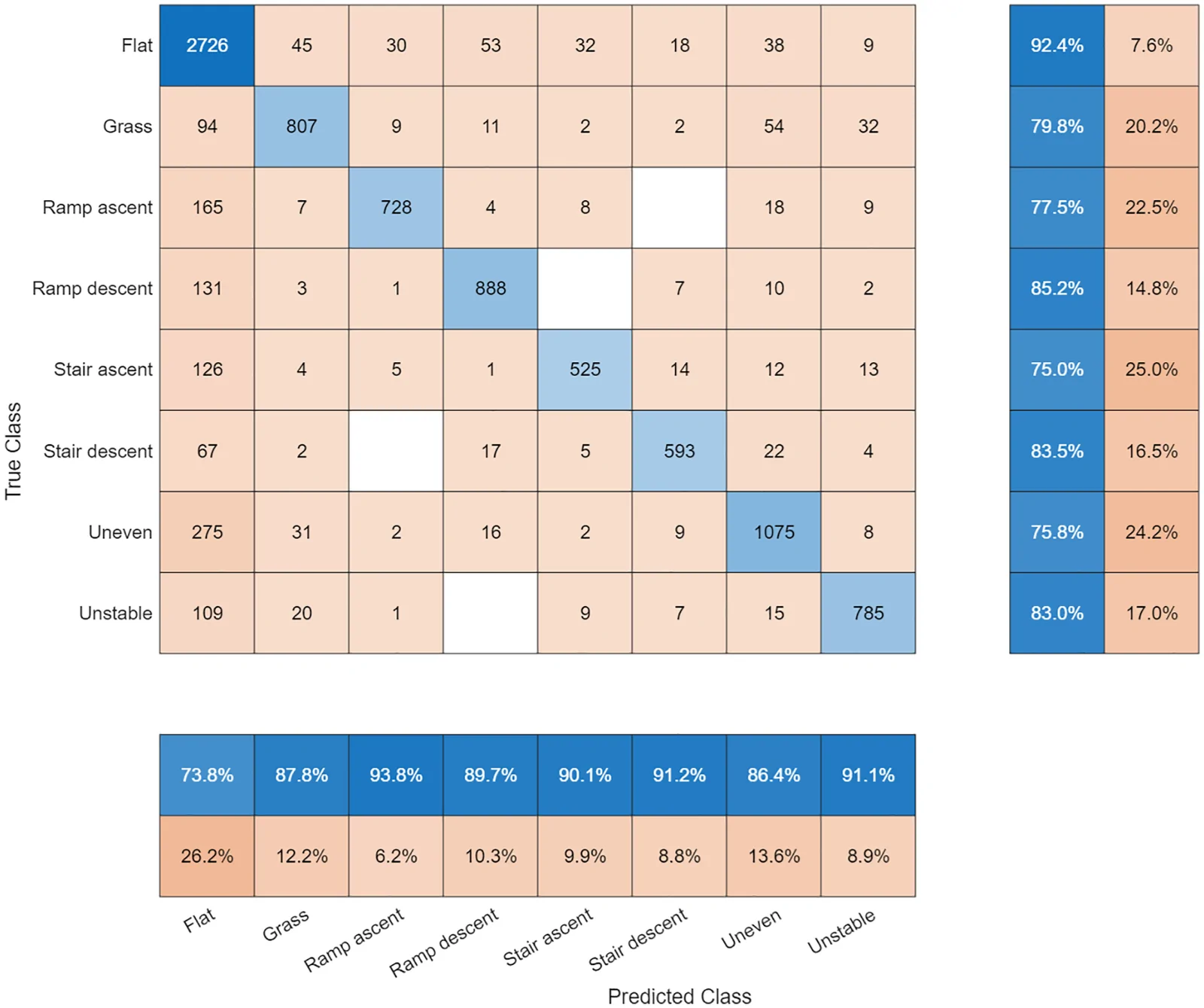
Confusion matrix for terrain classification RF Cross-validation.

These accuracies are similar to a previous study [19] which achieved a leave-some-out accuracy of 56.68% and a cross-validation accuracy of 78.46%, but flat, grass, uneven and unstable terrain were all grouped as flat terrain. Using the same terrain groupings our model achieved a leave-some-out accuracy of 79.05% (4.03) and a cross-validation accuracy of 90.21% (0.78). These accuracies are comparable to other studies that classified limited terrain in laboratory conditions [17,18,42]. Collecting data on different variations of the same terrain category should make the model more robust but limits number of strides on each variation which could be lowering the accuracies. The variation in the lower limb prosthetic [13] could also be contributing to lower accuracies. Although the dataset contained data from more lower limb prosthetic users than had been collected in previous studies, a larger dataset would be needed to achieve high accuracies and robustness for clinical use. Furthermore, the data used for algorithm development were collected in real-world environments under study conditions (with the researcher present); it has been shown that there can be changes in gait in study conditions compared to free-living behaviour [14]. Training the models on data collected in free-living conditions would further improve the robustness of the accuracies. The cross‑validation accuracies being significantly higher than leave‑some‑out accuracies indicates participant‑specific overfitting could be present. No class‑weighted training or additional regularisation was applied to the models, which could be addressed in future work.

In this study a ramp was classified as a surface that was steeper than 1:20, the UK building regulation definition of a slope [51], but this meant any surface less steep was classified as flat. It would be unreasonable to assume that a participant’s gait only changes on slopes steeper than 1:20 and that gait then doesn’t continue to change with steeper slopes. Strides on slopes just steeper than 1:20 could be similar to strides on slopes just shallower than 1:20 which could be a source of misclassification in the model. Moreover, what constitutes “uneven terrain” was decided by the researcher conducting the study. Even with terrain that was considered very uneven there could be places where the foot placement is flat, but for this study it would have been classified as uneven. This could be a source of lower accuracies when classifying uneven terrain as seen in the confusion matrices of Figs 3 and 4. Measuring ankle movement instead of classifying terrain could offer the required clinically relevant data more accurately.

In this paper transition steps (steps moving the person from one terrain to another) were not identified. Some studies have found transition steps to be different [17,18,42], and therefore they could affect accuracies if not labelled correctly. There is also the question of when a stride should be classified as a certain terrain. A stride was detected during the swing phase and the terrain the foot lands on during the stride was labelled, however, no analysis was done to identify which parts of the gait are the most important for the classifiers and therefore where the transition between different gait patterns for different types of terrain occurs. Labelling transition steps could eliminate this, or another option that was not explored would be using stride-based windows which contain multiple strides.

It must be noted that the final SVM LSO walking aid recognition accuracy of 61.56% was low considering that 61.00% of the steps were conducted without a walking aid, therefore classifying all steps as without a walking aid would produce a similar accuracy to the final model. The CV accuracy of 82.90% is also too low for clinical use, as the 17.10% error could inaccurately change a clinician’s assessment of a patient and affect the prosthetic components they are prescribed. A walking aid-mounted IMU may be a solution to enhance classification accuracy of walking aid use, however, it would require an additional sensor to the shank-mounted IMU, and more if the patient used multiple walking aids.

The clinical evaluation demonstrated the clinical relevance and desire for the data that the system outputs. Load through the prosthesis was highlighted as an additional measure that could be clinically beneficial. There have been studies [16,18,38,39] that have incorporated load through a prosthesis with data of an IMU to classify limited terrain in controlled conditions, and these indicate that a measure of load through a prosthesis could improve terrain classification. No previous study has investigated walking aid recognition from load through a prosthesis, but it has been shown that users can offload 25% [55] of body weight when using a walking stick and 12% using a walking frame [56], hence load measurement may also enhance walking aid use classification.

The limitations of the current system’s accuracies and robustness indicate further research is needed before such a system could be used in clinical practice. However, this study demonstrates the potential clinical impact a system could have pending further development and a larger training dataset.

A formal test–retest reliability assessment for stride detection, cadence estimation or IMU attachment repeatability on the prosthesis pylon in this specific cohort was not performed. Instead, this research relied on a previously validated stride‑detection method from previous work and standardised mounting procedures [6]. Future studies should quantify how repeated donning of the IMU and day‑to‑day variability in placement affect classification accuracy. Phase‑2 of the study only involved 4 clinical experts analysing reports on 3 prosthetic users. The small participant sample should be considered and caution taken when generalising the findings. Moreover, clinicians responded to simulated reports rather than using the system to make assessment decisions, so we cannot determine whether, or how, the information would actually change K‑level assignments or component selection in routine practice. A controlled study designed to test whether the reports alter K‑level assignment or component selection is an important next step.

This research did not establish accuracy thresholds clinicians would consider sufficient for K‑level assessments or prescription decisions. How specific errors might influence clinical decisions was also not investigated. In Phase 2, clinicians commented that inaccuracies could be alleviated by combining system outcomes with current assessment methods rather than replacing these. Future work should include prospective studies in which clinicians use system outputs in real prescription workflows, to determine acceptable accuracy levels and identify which types of errors are clinically tolerable, also under consideration of risks to safety.

## Conclusions

This study developed an original methodology for objectively determining activity levels from inertial sensor data. Through rigorous data collection and exhaustive algorithm design we were able to show that a single, prosthesis-mounted IMU has the potential to offer objective, real-world activity data to aid clinical decision-making for lower limb prosthetic users. The work was further substantiated by interviews which revealed that there is a desire from clinicians to obtain these data on their patients to improve and support K-level decisions. Further work could be conducted to incorporate an additional IMU on the walking aid and/or a measure of load through the prosthesis to improve walking aid use classification. A measure of load could furthermore be used to improve terrain classification and measure the stress on prosthesis components. The positive clinical evaluation alludes to the clinical significance of this technology to bridge the gap between subjective assessments and objective data for better patient outcomes in the continuously growing amputee population [1,2]. Future work needs to collect further critical data to pave the way to clinical deployment.

## Supporting information

S1 File Supplement with detailed qualitative interview results from “Lower limb prosthetic users’ activity classification through real-world inertial sensing”. (PDF)

## References

[1] WHO. WHO standards for prosthetics and orthotics. WHO and USAID. 2017.

[2] Ziegler-Graham K, MacKenzie EJ, Ephraim PL, Travison TG, Brookmeyer R. Estimating the prevalence of limb loss in the United States: 2005 to 2050. Arch Phys Med Rehabil. 2008;89(3):422–9. doi: 10.1016/j.apmr.2007.11.005 18295618

[3] Amputee-Coalition. Do you know your k-level? An interview with interim president CEO Sue Stout about the importance of amputees knowing their k-levels. inMotion. 2013.

[4] Balk EM, Gazula A, Markozannes G, Kimmel HJ, Saldanha IJ, Resnik LJ. Lower limb prostheses: measurement instruments, comparison of component effects by subgroups, and long-term outcomes. Rockville (MD): Agency for Healthcare Research and Quality (US). 2018. 30285344

[5] Jamieson AG, Murray L, Buis A. The use of physical activity outcomes in rehabilitation interventions for lower limb amputees: A systematic review. Can Prosthet Orthot J. 2020;3(1):33931. doi: 10.33137/cpoj.v3i1.33931 37614661 PMC10443482

[6] Wassall M. Advancing prosthetic prescriptions: developing a sensor-based system for objective activity assessment. University of Salford. 2025.

[7] Catalfamo P, Ghoussayni S, Ewins D. Gait event detection on level ground and incline walking using a rate gyroscope. Sensors (Basel). 2010;10(6):5683–702. doi: 10.3390/s100605683 22219682 PMC3247727

[8] Simonetti E, Villa C, Bascou J, Vannozzi G, Bergamini E, Pillet H. Gait event detection using inertial measurement units in people with transfemoral amputation: a comparative study. Med Biol Eng Comput. 2020;58(3):461–70. doi: 10.1007/s11517-019-02098-4 31873834

[9] Gouda A, Andrysek J. Rules-based real-time gait event detection algorithm for lower-limb prosthesis users during level-ground and ramp walking. Sensors (Basel). 2022;22(22):8888. doi: 10.3390/s22228888 36433483 PMC9693475

[10] Maqbool HF, Husman MAB, Awad MI, Abouhossein A, Mehryar P, Iqbal N, et al. Real-time gait event detection for lower limb amputees using a single wearable sensor. Annu Int Conf IEEE Eng Med Biol Soc. 2016;2016:5067–70. doi: 10.1109/EMBC.2016.7591866 28269407

[11] Gatti AA, Stratford PW, Brenneman EC, Maly MR. GT3X+ accelerometer placement affects the reliability of step-counts measured during running and pedal-revolution counts measured during bicycling. J Sports Sci. 2016;34(12):1168–75. doi: 10.1080/02640414.2015.1096018 26487374

[12] Madaoui L, Kerdjidj O, Kedir-Talha M. Design and implementation of IMU-based locomotion mode recognition system on Zynq SoC. Microprocessors and Microsystems. 2023;102:104927. doi: 10.1016/j.micpro.2023.104927

[13] Liu Y, An H, Ma H, Wei Q. Novel feature extraction and locomotion mode classification using intelligent lower-limb prosthesis. Machines. 2023;11(2):235. doi: 10.3390/machines11020235

[14] Papapicco V, Chen B, Munih M, Davalli A, Sacchetti R, Gruppioni E, et al. A Classification approach based on directed acyclic graph to predict locomotion activities with one inertial sensor on the thigh. IEEE Trans Med Robot Bionics. 2021;3(2):436–45. doi: 10.1109/tmrb.2021.3075096

[15] Vu HTT, Cao H-L, Dong D, Verstraten T, Geeroms J, Vanderborght B. Comparison of machine learning and deep learning-based methods for locomotion mode recognition using a single inertial measurement unit. Front Neurorobot. 2022;16:923164. doi: 10.3389/fnbot.2022.923164 36524219 PMC9745042

[16] Gonzales-Huisa OA, Oshiro G, Abarca VE, Chavez-Echajaya JG, Elias DA. EMG and IMU data fusion for locomotion mode classification in transtibial amputees. Prosthesis. 2023;5(4):1232–56. doi: 10.3390/prosthesis5040085

[17] Bruinsma J, Carloni R. IMU-based deep neural networks: Prediction of locomotor and transition intentions of an osseointegrated transfemoral amputee. IEEE Trans Neural Syst Rehabil Eng. 2021;29:1079–88. doi: 10.1109/TNSRE.2021.3086843 34097612

[18] Zheng E, Wan J, Gao S, Wang Q. Adaptive locomotion transition recognition with wearable sensors for lower limb robotic prosthesis. IEEE/ASME Trans Mechatron. 2024;29(1):279–89. doi: 10.1109/tmech.2023.3278315

[19] Jamieson A. Development of a clinically-targeted human activity recognition system to aid the prosthetic rehabilitation of individuals with lower limb amputation in free living conditions. 2021. http://purl.org/coar/resource_type/c_db06 10.3390/s21248377 PMC8704297 34960463

[20] Labarrière F, Thomas E, Calistri L, Optasanu V, Gueugnon M, Ornetti P, et al. Machine learning approaches for activity recognition and/or activity prediction in locomotion assistive devices-a systematic review. Sensors (Basel). 2020;20(21):6345. doi: 10.3390/s20216345 33172158 PMC7664393

[21] Wang D, Gu X, Yu H. A comparison of four neural networks algorithms on locomotion intention recognition of lower limb exoskeleton based on multi-source information. J Bionic Eng. 2023;21(1):224–35. doi: 10.1007/s42235-023-00435-w

[22] Luo S, Shu X, Zhu H, Yu H. Early prediction of lower limb prostheses locomotion mode transition based on terrain recognition. IEEE Sensors J. 2023;23(22):27941–8. doi: 10.1109/jsen.2023.3320274

[23] Yin S, Chen X, Ma T, Wang Y, Guo Y, Leng Y. Environmental Recognition and Multimode Continuous-Phase Control for a Powered Transfemoral Prosthesis. In: 2023 IEEE International Conference on Development and Learning (ICDL); 2023 9–11 Nov. 2023. https://ieeexplore.ieee.org/document/1234567

[24] Zhong B, Silva RL da, Tran M, Huang H, Lobaton E. Efficient environmental context prediction for lower limb prostheses. IEEE Trans Syst Man Cybern, Syst. 2022;52(6):3980–94. doi: 10.1109/tsmc.2021.3084036

[25] Qian Y, Chen C, Xiong J, Wang Y, Leng Y, Yu H, et al. Terrain-adaptive exoskeleton control with predictive gait mode recognition: A pilot study during level walking and stair ascent. IEEE Trans Med Robot Bionics. 2024;6(1):281–91. doi: 10.1109/tmrb.2024.3349624

[26] Cheng S, Laubscher CA, Best TK, Gregg RD. Ambilateral activity recognition and continuous adaptation with a powered knee-ankle prosthesis. IEEE Trans Robot. 2025;41:2251–67. doi: 10.1109/tro.2025.3539206 40687340 PMC12269649

[27] Gao S, Wang Y, Fang C, Xu L. A smart terrain identification technique based on electromyography, ground reaction force, and machine learning for lower limb rehabilitation. Appl Sci. 2020;10(8):2638. doi: 10.3390/app10082638

[28] Li J, Zhang J, Li K, Cao J, Li H. A multimodal framework based on deep belief network for human locomotion intent prediction. Biomed Eng Lett. 2024;14(3):559–69. doi: 10.1007/s13534-024-00351-w 38645596 PMC11026357

[29] Son C-S, Kang W-S. Multivariate CNN model for human locomotion activity recognition with a wearable exoskeleton robot. Bioengineering (Basel). 2023;10(9):1082. doi: 10.3390/bioengineering10091082 37760184 PMC10525937

[30] Wang X, Bai S, Li L, Li Y, Yu H. Enhancing Locomotion-Mode Recognition and Transition Prediction With (Bio)Mechanical Sensor Fusion for Intelligent Prosthetic Knees. IEEE J Biomed Health Inform. 2025;29(11):7955–68. doi: 10.1109/JBHI.2025.3583319 40560708

[31] Ahkami B, Kristoffersen MB, Ortiz-Catalan M. Real-time locomotion mode detection in individuals with transfemoral amputation and osseointegration. J Neuroeng Rehabil. 2025;22(1):142. doi: 10.1186/s12984-025-01672-2 40551132 PMC12186424

[32] Gao F, Liu G, Liang F, Liao W-H. IMU-based locomotion mode identification for transtibial prostheses, orthoses, and exoskeletons. IEEE Trans Neural Syst Rehabil Eng. 2020;28(6):1334–43. doi: 10.1109/TNSRE.2020.2987155 32286999

[33] Mai J, Xu D, Li H, Zhang S, Tan J, Wang Q. Implementing a SoC-FPGA Based Acceleration System for On-Board SVM Training for Robotic Transtibial Prostheses. In: 2018 IEEE International Conference on Real-time Computing and Robotics (RCAR); 2018 1–5 Aug. 2018.

[34] Shell CE, Klute GK, Neptune RR. Identifying classifier input signals to predict a cross-slope during transtibial amputee walking. PLoS One. 2018;13(2):e0192950. doi: 10.1371/journal.pone.0192950 29451922 PMC5815617

[35] Hu B, Li S, Chen Y, Kavi R, Coppola S. Applying deep neural networks and inertial measurement unit in recognizing irregular walking differences in the real world. Appl Ergon. 2021;96:103414. doi: 10.1016/j.apergo.2021.103414 34087702

[36] Lu Z, Narayan A, Yu H. A Deep Learning Based End-to-End Locomotion Mode Detection Method for Lower Limb Wearable Robot Control. In: 2020 IEEE/RSJ International Conference on Intelligent Robots and Systems (IROS), 2020. 4091–7. 10.1109/iros45743.2020.9341183

[37] Lum PS, Shu L, Bochniewicz EM, Tran T, Chang LC, Barth J. Improving accelerometry-based measurement of functional use of the upper extremity after stroke: machine learning versus counts threshold method. Neurorehabil Neural Repair. 2020;34(12):1078–87. 10.1177/1545968320962483 PMC7704838 33150830

[38] Choudhary R, Gianey HK. Comprehensive review on supervised machine learning algorithms. In: International Conference on Machine Learning and Data Science (MLDS); 2017 14–15 Dec. 2017.

[39] Oh H-W, Hong Y-D. Divergent component of motion-based gait intention detection method using motion information from single leg. J Intell Robot Syst. 2023;107(4). doi: 10.1007/s10846-023-01843-0

[40] Chauhan P, Singh AK, Raghuwanshi NK. Classifying the walking pattern of humans on different surfaces using convolutional features and shallow machine learning classifiers. Proceedings of the Institution of Mechanical Engineers, Part C: J Mech En Sci. 2024;238(18):8943–54. doi: 10.1177/09544062241247956

[41] Qi Z, Song Q, Liu Y, Guo C. FSM-HSVM-based locomotion mode recognition for exoskeleton robot. Appl Sci. 2022;12(11):5483. doi: 10.3390/app12115483

[42] Marcos Mazon D, Groefsema M, Schomaker LRB, Carloni R. IMU-based classification of locomotion modes, transitions, and gait phases with convolutional recurrent neural networks. Sensors (Basel). 2022;22(22):8871. doi: 10.3390/s22228871 36433469 PMC9698430

[43] Shin D, Lee S, Hwang S. Locomotion mode recognition algorithm based on Gaussian mixture model using IMU sensors. Sensors. 2021;21(8):2785. 10.3390/s21082785 PMC8071300 33920969

[44] Reznick E, Embry KR, Neuman R, Bolívar-Nieto E, Fey NP, Gregg RD. Lower-limb kinematics and kinetics during continuously varying human locomotion. Sci Data. 2021;8(1):282. doi: 10.1038/s41597-021-01057-9 34711856 PMC8553836

[45] Jamieson A, Murray L, Stankovic L, Stankovic V, Buis A. Human activity recognition of individuals with lower limb amputation in free-living conditions: A pilot study. Sensors (Basel). 2021;21(24):8377. doi: 10.3390/s21248377 34960463 PMC8704297

[46] Antos SA, Danilovich MK, Eisenstein AR, Gordon KE, Kording KP. Smartwatches can detect walker and cane use in older adults. Innov Aging. 2019;3(1):igz008. doi: 10.1093/geroni/igz008 31025002 PMC6476414

[47] Moder T, Wisiol K, Wieser M. Walking aid identification using wearables. Cham: Springer International Publishing. 2016.

[48] Tazawa M, Arii H, Ibe Y, Kobayashi H, Chikuda H, Wada N. Effects of walking with a cane on frontal plane hip joint loading in patients with late-stage unilateral hip osteoarthritis. Arch Rehabil Res Clin Transl. 2022;4(3):100209. doi: 10.1016/j.arrct.2022.100209 36123985 PMC9482035

[49] Zhang Y, Tao C, Zhang X, Guo J, Fan Y. Effects of cane use on the kinematic and kinetic of lower-extremity joints in inexperienced users. J Biomech. 2023;146:111426. doi: 10.1016/j.jbiomech.2022.111426 36608543

[50] Hsue B-J, Su F-C. Gait and kinematics of the trunk and lower extremities in stair ascent using quadricane in healthy subjects. Gait Posture. 2009;29(1):146–50. doi: 10.1016/j.gaitpost.2008.06.001 18676145

[51] HM-Goverment. Protection from falling, collision and impact: Approved Document K. 2013.

[52] Huang L, Qin J, Zhou Y, Zhu F, Liu L, Shao L. Normalization techniques in training DNNs: methodology, analysis and application. IEEE Trans Pattern Anal Mach Intell. 2023;45(8):10173–96. doi: 10.1109/TPAMI.2023.3250241 37027763

[53] Hwang S, Kim J, Yang S, Moon HJ, Cho KH, Youn I. Machine learning based abnormal gait classification with IMU considering joint impairment. Sensors. 2024;24(17):5571. doi: 10.3390/s24175571 PMC11397963 39275482

[54] Braun V, Clarke V. Using thematic analysis in psychology. Qualitative Res Psychology. 2006;3(2):77–101. doi: 10.1191/1478088706qp063oa

[55] Youdas JW, Kotajarvi BJ, Padgett DJ, Kaufman KR. Partial weight-bearing gait using conventional assistive devices. Arch Phys Med Rehabil. 2005;86(3):394–8. doi: 10.1016/j.apmr.2004.03.026 15759217

[56] Thies SB, Bevan S, Wassall M, Shajan BK, Chowalloor L, Kenney L, et al. Evaluation of a novel biomechanics-informed walking frame, developed through a Knowledge Transfer Partnership between biomechanists and design engineers. BMC Geriatr. 2023;23(1):734. doi: 10.1186/s12877-023-04443-7 37957568 PMC10642022

