# Supplementary material for: Lower limb prosthetic users’ activity classification through real-world inertial sensing
Source: PLoS One. 2026 Oct 9;21(10):e0358882. doi: 10.1371/journal.pone.0358882 (PMC13652245; doi:10.1371/journal.pone.0358882)
Supplement: Supplementary file 1 [file pone.0358882.s001.pdf]

Supplement with detailed qualitative interview results from "Lower limb prosthetic users' activity classification through real-world inertial sensing"

Matthew Wassall[1], Malcolm Granat2, Saeed Zahedi[3], Sibylle Thies[2]

[1] Department of Neuromedicine and Movement Science, Norwegian University of Science and Technology, Trondheim, Norway

[2] School of Health & Society, University of Salford, Salford, United Kingdom

[3] Blatchford Prosthetics, United Kingdom

OVERVIEW

Semi-structured interviews were conducted with four clinical experts to investigate the clinical relevance of the IMU system. To be able to demonstrate the capabilities of the system, three lower limb prosthetic users were first monitored with the system over a two-week period. The prosthetic users' data were then classified using the classification models. To investigate whether the system data may provide additional insights to those obtained through standard clinical assessments, each prosthetic user was also assessed by a clinician using a standard K level assessment. The interviewees were then presented with a summary of the clinical assessment and a short report that summarised the system output for each participant at least 24 hours before the interview took place. The study had university ethical approval (4743), and all participants provided informed consent.

METHODS

Clinician Participants

The inclusion criteria for the interviewees were: experience with activity level assessments in users of lower limb prostheses, able to provide informed consent, and able to do an interview over the phone in the English language. Four clinical experts were recruited who all had between 16 and 27 years' clinical experience. Two worked in the NHS and two in private clinics.

Clinicians were interviewed in relation to the reports complied for the 3 prosthetic users that contained their K level and also the IMU-derived activity outcomes (see details below). An interview guide, developed with influence from work of Kalio et al. (1) was sent to all clinician participants before the interviews to give them an understanding of the type of questions that would be asked and why. All interviews were conducted via video call and audio was recorded using an external General Data Protection Regulation (GDPR) compliant recording device. The audio was then transcribed, and any identifying details of the participants were removed from the transcripts. The recordings were transcribed using speech to text software and then manually checked and corrected where needed. The transcripts were analysed using thematic analysis based on a framework approach described by Braun and Clarke (2). Initial coding was conducted, iteratively developing a

coding framework that was reviewed and refined. Codes were then grouped into higher-level themes through repeated reading and comparison across transcripts. Given the small number of interviews (n=4), formal inter-rater reliability statistics were not calculated.

Prosthetic User Participants

Three lower limb prosthetic user participants were recruited that had taken part in the previous data collection that was used to develop the models. All three were male and above 60 years old (mean±SD age: 70±11.4 years). Two were transtibial and one was transfemoral. They provided the data for the reports that were subsequently discussed with the clinician participants. Specifically:

An independent clinician assigned K levels to the 3 prosthesis users as per standard practice. The assessments were conducted at the University of Salford in the prosthetic and orthotic clinic room, to be as similar as possible to current clinical practice. The clinician had 8 years of experience conducting K level assessments for prosthetic component prescription. The assessments were twenty minutes long, as this was the same length as the clinician would usually have to conduct clinical assessments. The assessments were conducted as close to a clinical assessment as possible, and consisted of the clinician having a conversation with the prosthetic user about their activity and medical history, and ended with the clinician deciding on an appropriate K level for each participant. A summary of their clinical assessment that included the information that the clinician deemed key for deciding each participant's K level made up the first part of the report that was then discussed with the clinician participants.

The second part of the report contained a summary of the data that included the average daily steps in total and on each terrain, the average recorded cadence and a percentage of the steps that were at a cadence 20% higher than the average, and the percentage of steps that used a walking aid. Furthermore, a table displayed data for each day of the data collection period, and this table included the number of steps on each terrain and in total, the average daily cadence and the percentage of steps that day that were at a cadence 20% higher than the participant's average cadence, and the number of steps taken with and without a walking aid plus the percentage of steps taken with a walking aid.

The reports containing K Level information and activity data are shown below for each of the prosthetic patient participants. Some information has been retracted or made less identifiable to ensure participants can not be identified from this published report.

Prosthesis User 1

Clinical assessment summary

- Transtibial Left leg
- Cause: Trauma
- 60-70 years old
- Hobbies include walking (approximately 4 to 5 miles a day)
- Carer for family member
- Does not play sport
- Does not smoke
- Drinks a bit
- Type 2 diabetes
- Skin condition is good
- Oxford scale 5 for muscle strength in amputated limb
- No knee instability
- Walks unaided
- Is able to don and doff their prosthetic unaided

Assessed as a K3

Summary of recorded data

On average: **7905 steps per day**
Average steps on **grass** a day: **672**
Average **stairs climbed** a day: **254**
Average **stairs descended** a day: **132**
Average steps **up a slope** a day: **614**
Average steps **down a slope** a day: **976**
Average steps on **uneven terrain** a day: **205**
Average steps on **unstable terrain** a day: **244**
Average cadence: **73.9 Steps per minute**
% of steps **above 20% average Cadence**: **27.4%**
% of walking aid use: **0%**

	Day 1	Day 2	Day 3	Day 4	Day 5	Day 6	Day 7	Day 8	Day 9	Day 10	Day 11	Day 12	Day 13	Day 14	Day 15
Flat	512	2418	5242	6788	6854	2926	7174	2682	5822	4392	6238	4636	5886	6440	4112
Grass	74	282	424	474	1232	706	978	324	1690	344	1156	786	516	592	496
Up Stairs	92	506	254	370	168	160	174	190	244	308	336	210	214	396	174
Down Stairs	8	32	172	240	164	42	66	60	152	266	174	146	234	112	120
Up Slope	2426	426	62	106	212	1352	1162	172	788	62	1074	298	84	476	512
Down Slope	470	3868	2520	1012	1020	818	1436	464	910	194	340	362	336	560	336
Uneven	10	48	210	308	668	176	522	22	252	82	74	220	162	130	194
Unstable	106	374	492	100	170	178	344	78	280	126	468	152	174	324	288
**Total**	**3698**	**7954**	**9376**	**9398**	**10488**	**6358**	**11856**	**3992**	**10138**	**5774**	**9860**	**6810**	**7606**	**9030**	**6232**
Cadence steps per minute	65.1	73.9	72.3	73.6	74.4	75.6	79	74.7	77	67.3	72.1	71.5	70.3	74.2	72.3
% above 20% normal Cadence	28.2	3.2	25.6	25.2	29.7	33.4	40.4	35.7	32.4	21.8	31.3	23.8	21.3	30.6	21.5
Without a walking aid	1849	3976	4688	4699	5244	3179	5928	1996	5069	2883	4930	3404	3803	4515	3116
With a walking aid	0	1	0	0	0	0	0	0	0	4	0	1	0	0	0
% walking aid use	0%	0%	0%	0%	0%	0%	0%	0%	0%	0%	0%	0%	0%	0%	0%

Prosthesis User 2

Clinical assessment summary

- Transtibial left leg
- Cause: Trauma
- 80-90 years old
- Has a pacemaker
- Has high blood pressure
- Has asthma
- Has type 2 diabetes
- Does not smoke
- Drinks a bit
- Walks daily (approximately 3 to 4 miles)
- Hip replacement in contralateral leg
- No pain in contralateral leg
- Reddening of skin at end of residual limb and tibia head
- Walks unaided
- Is able to don and doff their prosthetic unaided

Assessed to be a K3, but noted that current prescription is a K2 leg

Summary of recorded data

On average: **6562 steps per day**

Average steps on **grass** a day: **967**

Average **stairs climbed** a day: **133**

Average **stairs descended** a day: **66**

Average steps **up a slope** a day: **585**

Average steps **down a slope** a day: **960**

Average steps on **uneven terrain** a day: **419**

Average steps on **unstable terrain** a day: **166**

Average cadence: **91.8 Steps per minute**

% of steps above **20% average Cadence**: **16.5%**

% of walking aid use: **0%**

	Day 1	Day 2	Day 3	Day 4	Day 5	Day 6	Day 7	Day 8	Day 9	Day 10	Day 11	Day 12	Day 13	Day 14	Day 15
Flat	3604	3088	3614	2758	2412	4066	3222	2582	3654	2588	4258	2632	3314	2216	4954
Grass	396	850	296	1288	1610	660	538	954	1544	1228	436	1874	1084	856	886
Up Stairs	132	106	202	126	150	120	90	122	204	76	170	128	136	80	158
Down Stairs	72	62	126	72	66	30	52	60	74	60	102	50	50	28	86
Up Slope	604	516	286	730	632	866	820	624	642	552	290	418	652	652	498
Down Slope	332	712	216	1150	1108	956	966	822	1136	1666	1158	1162	1416	1170	436
Uneven	260	462	102	364	478	264	380	250	1124	470	266	788	506	288	290
Unstable	142	184	136	112	218	102	214	118	270	146	222	152	188	68	220
**Total**	**5542**	**5980**	**4978**	**6600**	**6674**	**7064**	**6282**	**5532**	**8648**	**6786**	**6902**	**7204**	**7346**	**5358**	**7528**
Cadence steps per minute	95.2	85.8	84.5	87.4	87	94.5	91.1	91.8	96.2	96.4	90.4	93.3	92.6	98.5	90.5
% above 20% normal Cadence	18.4	6.1	0.8	5.4	7.6	16.6	8.7	20.2	25.2	22.2	30.7	17.4	11.4	30.4	21.5
Without a walking aid	2771	2990	2489	3300	3337	3532	3141	2766	4324	3393	3451	3602	3673	2679	3764
With a walking aid	0	0	0	0	0	0	0	0	0	0	0	0	0	0	0
% walking aid use	0%	0%	0%	0%	0%	0%	0%	0%	0%	0%	0%	0%	0%	0%	0%

Prosthesis User 3

Clinical assessment summary

- Transfemoral left leg
- Cause: Compartment syndrome
- 60-70 years old
- Has type 2 diabetes
- Does not smoke
- Drinks a little
- Lives alone
- Can walk 11Km, regularly walks 5Km
- Does not run and no desire to
- Cycles 12 to 15 miles a day
- Goes to the gym regularly
- Coaches and plays semi-active sport

Assessed to be a K4

During assessment had a fall that restricted normal activities.

Summary of recorded data

On average: **4032 steps per day**
Average steps on **grass** a day: **208**
Average **stairs climbed** a day: **131**
Average **stairs descended** a day: **12**
Average steps **up a slope** a day: **482**
Average steps **down a slope** a day: **364**
Average steps on **uneven terrain** a day: **35**
Average steps on **unstable terrain** a day: **23**
Average cadence: **73.4 Steps per minute**
% of steps above **20% average Cadence**: **23.8%**
% of walking aid use: **1%**

	Day 1	Day 2	Day 3	Day 4	Day 5	Day 6	Day 7	Day 8	Day 9	Day 10	Day 11	Day 12	Day 13	Day 14
Flat	3492	6692	1770	1944	1500	1684	2462	1252	1402	946	4174	5592	2680	3302
Grass	194	234	142	256	4	164	162	92	82	118	322	344	354	438
Up Stairs	36	1236	50	56	78	60	48	52	38	14	42	36	58	30
Down Stairs	10	50	18	14	4	12	8	12	20	8	4	4	6	4
Up Slope	522	1142	162	208	58	206	212	46	132	78	1064	1578	438	902
Down Slope	518	1088	120	270	24	180	122	42	152	86	510	1292	136	550
Uneven	64	22	18	98	8	16	20	8	24	12	46	64	68	22
Unstable	30	46	18	28	34	20	16	28	10	8	32	18	26	6
**Total**	**4866**	**10510**	**2298**	**2874**	**1710**	**2342**	**3050**	**1532**	**1860**	**1270**	**6194**	**8928**	**3766**	**5254**
Cadence steps per minute	75.9	77.9	59.1	66.8	65.1	60.3	59.7	54.4	67.7	61.7	78.8	83.5	67.9	80.4
% above 20% normal Cadence	19.6	28.7	8.9	11.8	17.3	9.7	5.0	3.8	24.3	1.9	3.6	54.5	12.6	40.9
Without a walking aid	2432	5247	1129	1428	838	1151	1500	733	924	629	3095	4464	1874	2626
With a walking aid	1	8	20	9	17	20	25	33	6	6	2	0	9	1
% walking aid use	0%	0%	2%	1%	2%	2%	2%	4%	1%	1%	0%	0%	1%	0%

Results

Thematic analysis of the interview transcripts revealed the following six themes: "General feedback" and "Clinical use", "Presentation of system output", "Comparison of system data with clinical assessments", "Areas for improvement", with some of these containing a number of subthemes.

Theme 1: General feedback on the system

This theme had two subthemes, namely "Positives" and "Output data of the system" which are discussed in the following two sections.

Positives

When asked about their general thoughts on the data that the system produced, the interviewees gave very positive responses:

"I found the data very helpful." (C1)

"I thought it was really good, honestly, really, the thing is, good stuff." (C2)

"I think it's amazing data. It's really, really impressive." (C4)

C4 even inquired about when the system would be available for clinical use:

"Yeah, when can we use this? It's Fantastic, it's really good." (C4)

Output data of the system

Delving deeper and asking about the measures that the system produced, the interviewees were happy with the measures and thought they were all clinically relevant:

"Those kind of core skills are really the key ones that we focus on clinically, and they are some of the ones that people identified as important when we did our research about important outcome domains." (C1)

"No, there's nothing there that I think why? Why is that there, no, all of that is everything in there is what we'd want to know." (C3)

Initially when asked if there were any other measures that they thought would improve the clinical relevance, two of the interviewees said that there was nothing they could think of:

"There's nothing screaming out there that I'd think, Oh, we're missing." (C3)

"I don't think there's anything else that we would want to know. I don't think so." (C4)

However, there were other measure that these interviewees mentioned later as the interview progressed, namely force/load and temporal measures.

Regarding force/load measurement, three reasons were given as to why including load through the prosthesis would aid in clinical decision making. The **first** reason provided was that some manufacturers specify that a K4 prosthetic should be given if the user is putting high stress through the prosthesis, e.g.:

"K4 you're then saying exhibiting high impact stress or energy levels." (C1).

The **second** reason provided was to see if the user is putting 50% of their body weight through their prosthesis:

“To see actually they are putting 50% of the body weight through the prosthetic.” (C3)

This was viewed as a means to reduce the risk of long-term health issues relating to uneven gait:

“In terms of protection of the body.” (C3)

The **third** and final reason given to support a need for load measurement was to see if the user is not only offloading their prosthesis but also has the ability to put additional load through it:

“Taking some weight off, you're offloading your leg.” (C4)

“A lot of them will say that they will carry things.” (C4)

Regarding measures relating to wear time and active time were also said to be of interest.

“One of the things that I've always found quite interesting is how long someone wears a device.” (C2)

With one reason for this being to better understand how much a user uses their prosthesis, which is a criterium for prescription:

“A lot of the criteria for different prescriptions {are} around full-time users.” (C4)

And this interviewee further emphasised that there remains uncertainty about what their patients are saying:

“If they say that they wear their leg all day, but actually the reality is they just leave it against a wall all day…” (C4)

Three of the interviewees stated that active time would be useful not only to judge active periods but also periods of inactivity:

“If you could determine standing active time, where they're walking, and how much the leg is used from, from morning to night, then it would help with getting prescriptions right.” (C2)

“It's good to know the periods of inactivity, and how long they're wearing the leg for.” (C3)

“inactivity would be useful.” (C4)

In summary, clinicians provided overwhelmingly positive feedback on the system’s output, particularly highlighting its clinical relevance and the utility of the data it generates. One clinician even enquired about the system's availability for widespread use. The only additional measures the participants brought up that would be desired clinically were load through the prosthesis, wear time and active time. Active time could already be obtained from the current data, by quantifying stepping periods. The load through the prosthesis could not be obtained with just an IMU, for such an additional sensor would need to be integrated. Measuring the load through the prosthesis was discussed in the main paper also as a way of improving walking aid recognition. Load through the prosthesis could further serve as an accurate way of measuring wear time.

Theme 2: Clinical use

The interviewees further considered how the system could be used clinically, and how it would be helpful for their clinical decision making. Their answers regarding this theme could be categorised into three subthemes: 1) supporting current practice, 2) presentation of system output, 3) impact of the system on current practice, and 3) benefit of additional information about patients.

Supporting current practice

Three of the interviewees talked about how the system data could be used to support current practice by proving and justifying clinical decisions, enabling more in-depth conversations on patients' lives, and proving deeper insights into their patients' lives.

"I think it actually proves the K levels. It helps you actually have a data driven way of assessing someone's K levels as just now, it's a 20-minute interview." (C2)

"Yeah, because then it can support *{clinical decision making}*." (C3)

"I think it definitely has a place. And I think especially when we are being asked to justify why we're prescribing specific things." (C4)

One reason why they thought the system data would help is because of the subjectiveness of their current assessments:

"It's always good to know extra data with prosthetics, because we're always lacking a little bit behind everywhere else, aren't we, because it's, it's so subjective." (C3)

C3 expanded on the subjectiveness by adding that this can mean that sometimes the clinician cannot fully believe what their patients are saying:

"The patient walks approximately four to five miles a day. We're just going off them saying that." (C3)

C4 gave a reason for why being able to justify prescriptions would be beneficial to a clinic, i.e. because of the cost of prothesis components:

"It would be more frequently used than you can imagine, because you're talking about money here, talking about spending money, saving money, justifying prescriptions." (C4)

They also commented on how the system could be used practically:

"It would allow us to have a much fuller conversation about why we're going to change their prescription." (C4)

Three of the interviewees further talked about how the system data could change the assessments by facilitating in-depth conversations about their patients' activities:

"Often they come in and you say, kind of how do you spend your time? And they go, I like to watch a bit of telly. And then you're like, come on, I need more than that. But it's really hard to get it out. And then, whereas this could help really make those conversations more specific." (C1)

"You can then go into it and say, Well, what, what were you doing on this day that you can, that allowed you to achieve that many and why in the days?" (C3)

"More prompts, more information, to dig a little bit deeper. And it also does affect the ability to justify the prescription." (C4)

“We say to people, you know, do you go up down slopes? That's kind of it. We don't really go into the detail of what type of slope, what kind of surfaces? Is it grass? Is it gravel? Has It got a rail? Whereas this would give you a bit more of a prompt to ask the detail around.” (C4)

C4 gave a bit more context on why current assessments don’t always obtain these details whereas having the system data could:

“It's not necessarily that we don't want to know these things. I think we just have so many other things to ask them. I think this would allow us to kind of go, okay, well, talk me through, what does that slope look like?” (C4)

And that the sensor data could make having difficult conversations easier:

“I think data, in my mind is always that ability to actually have those conversations without it becoming very personal, you know, feelings and all that kind of stuff. At least, you can kind of use the data to kind of have the kind of more difficult conversation.” (C4)

C1 also highlighted that the questions prompted by the sensor data could also verify the sensor data:

“You'd be able to pull out some of the inaccuracies in data by verifying it with the patient as best you could.” (C1)

C1 discussed how the system data could give more detail about their patients’ lives that could affect clinical decisions:

“It can direct those conversations to really unpick what's going on for the patient, and that then affects your clinical decision making, because ultimately, you're trying to match your products to the patient's life.” (C1)

Notably, C4 suggested that the objective system data could potentially be more accurate than insights gained through subjective assessments during a single clinic visit:

“I would say it's much, much more accurate than what we currently have by far.” (C4)

And the justification for this was that the system can collect data for two weeks whereas current assessments happen at one specific time:

“A lot of the K levels are based on the way that they walk into the building, along with kind of what they say they're doing, the physio team will do an amputee mobility predictor, but that is one point in time. And the problem with that is, if they're having a bad day, then it's bad. If they're having a good day, is good, but it's not consistent.” (C4)

In summary, the clinical experts felt the system data could be used to support current assessments to justify decisions, facilitate conversations that provide more detail about their patients’ lives, help with difficult conversations and to obtain more information about their patients’ activities. This would make prescriptions more objective and help clinicians be more confident in making clinical decisions.

Theme 3: Presentation of system output

How the system data were presented was also discussed. All of the interviewees gave generally positive feedback on how the data were presented:

“I liked the summary, that was really useful.” (C1)

“It was nice and simple, was dead straight forward so you can easily print off and stick in a patient's notes.” (C2)

“It's good to have it broken down” (C3)

“Summary I quite like.” (C4)

C1 and C2 highlighted the percentage of strides above 20% normal cadence as an aspect they both liked:

“The other thing I liked was percentage of steps above 20% average cadence, because that was quite useful to think about variable cadence.” (C1)

“You've got the percentage of steps above cadence. I think that is a nice thing to be able to ask a question about, okay, so you walked a wee bit faster. Do you remember what it was you were doing.” (C4)

There were three suggestions made by the participants to improve the way the data is displayed. These were having the data visualised in charts/graphs, having normative data and having percentages instead of just numbers.

Regarding the former, two of the interviewees commented on how the table with the daily data may not get read:

“It's not exciting to look at.” (C3)

“A lot of numbers in a table like that could be maybe not confusing, but it's just harder to read.” (C4)

C4 also justified this by pointing out that a clinical team might not have the time to decipher the table:

“I just know that the clinical team will probably not spend the time that they should do to actually try and understand that.” (C4)

C2 suggested a bell curve for the cadence of different steps:

“If you can get a bell curve that would show how much time or how many steps a day on average, they took at different walking speeds.” (C2)

C4 suggested graphs might help but wasn’t completely confident with the suggestion:

“It's almost like you want to have, a kind of graph, but I don't know if people would be off be put off by that.” (C4)

But they did make it known that it would be beneficial to change how the table is currently displayed:

“I feel like it needs to get split up. It's quite a lot to look at.” (C4)

Regarding inclusion of normative data, two of the interviewees thought that typical values for each K level would be helpful:

“Normative data is really useful.” (C1)

“I don't know if you can do this, is to see, is there a normal value.” (C4)

C4 expanded on this comment by explaining that the clinician might be comparing values against their own measures and not typical prosthetic users:

“I think as a clinical team, it would be difficult to know if that was good or bad, because they'll be basing it on their own step counts, which is unrealistic. So, a guy, for example, would know that actually 6500 steps a day is pretty good for an amputee, but I would think a lot of my clinical team might think that's pretty poor.” (C4)

Further regarding the presentation of the data, C4 thought it would be easier to read the daily table if some of the numbers were percentages of daily steps instead of just numbers:

“A percentage. Because, quite honestly, if you're doing the table, you can see quite clearly how many steps go up down each day. So maybe it's more of a percentage rather than a proportion, rather than a number.” (C4)

In summary, all the participants liked the summary of the system’s data they had been presented with, but some of the participants thought the table of daily activities might not be easy to read, especially for clinicians. Visualizing the data might help, with the use of charts and graphs being suggested, and alternatively percentages of daily steps taken under the various conditions might also make the data easier to understand. Normative data were also suggested, but before provision of such will become possible more data needs to be collected in the real-world.

Theme 4: Comparison of system data with clinical assessments

The interviewees were asked to examine the system’s data presented on the lower limb prosthetic users and compare it with the outcomes of the standard clinical assessments.

For prosthesis user 1, C1 said that the system data might have changed the prescription for the participant, raising their K level to a K4:

“For *{prosthesis user}* 1, when I looked at his clinical assessment, I felt like he was a K3, so I thought that matched well. But then, when I looked at his summary data, he seemed really active. So, he was like some days he was right up on 10,000 steps a day, and most days he was hitting that kind of six to 8000 that you might expect for people with a disability. He did a lot of stairs. He did a lot of uneven ground and a lot of slope, and so it made me think about whether he should have actually been a K4 user.” (C1)

They further elaborated:

“That kind of data. So, the total number of steps, the steps on different surfaces and during different activities, and the number and the percentage of cadence made me think he should have been a K4 which is interesting, because clinically I wouldn't have put him into K4, so that was that one.” (C1)

Contrary to this, interviewees C2 and C4 both thought the step count for prosthesis user 1 was low in relation to the clinical assessment outcomes:

“Just seemed to me to be quite low.” (C2)

“I think it says 4 to 5 miles a day. And so, I think the sensor data seems to be a wee bit lower than what the patient reports. But I would have thought that four to five miles a day would have been more than 7900 steps.” (C4)

For prosthesis user 2, interviewee C1 thought the system data matched the clinical data:

“Participant *{prosthesis user}* 2, I thought, was pretty straightforward. Like, his clinical assessment and his data, I thought, like were kind of reasonably well matched.” (C1)

Whereas C3 was surprised that the prosthesis user was as active as they were from the system data compared to the clinical assessment outcomes:

“Participant *{prosthesis user}* 2, you wouldn't have necessarily predicted him to be as active because of his condition and his age compared to participant *{prosthesis user}* 1, but that, you know, it shows that an 86 year old with a load of comorbidities, hip replacements and everything, can be as active as other people. And I think maybe sometimes we underestimate an aging population. So, I think that's quite useful to see that this man is, obviously a very active man, despite everything else.” (C3)

For prosthesis user 3, the interviewees acknowledged that the participant’s fall might have affected the recorded data:

“I probably would have put him as a K3, but it's possible that his fall could have made his activity data look worse than it was.” (C1)

Interviewee C3 commented on the same prosthesis user that their step count was low compared to the clinical assessment outcomes:

“Participant *{prosthesis user}* 3, yeah, reading, what? Reading, you know, type two diabetes, compartment syndrome, after a fall, doesn't smoke, drinks, lives alone, walks 11 kilometres regularly, walks five doesn't run, but cycles a lot, goes to the gym, but then looking at his step count, it's lower than I would have thought it would be.” (C3)

But said that, the breakdown of daily activities was able to help explain this:

“Some days his, his total step over 10,000 and then other days, it'd be 1500.” (C3)

And this was perceived to potentially help direct clinical assessments to understand why there are inconsistencies:

“It's good to have it broken down, and then you can then go into it and say, well, what, what were you doing on this day that you can, that allowed you to achieve that many and why in the days?” (C3)

Interviewee C1 also commented on how the system data could change the prescriptions they would give the participants:

“I just had the clinical and then actually, I'm looking at the summary data and thinking, oh, my judgments, my assumptions, based on kind of all that clinical data might not actually be that accurate as to how these people live their lives. So, the two together was a really nice combination to help make a decision.” (C1)

They also remarked that it might make them think about what additional support the prosthesis users might need in addition to just prosthetic components:

“It would also really make me think about the types of prosthesis. Because if he's doing like, he's doing quite a lot of like, if they're doing quite a lot of slope walking, it's like, does he live on a hill? Have we got them on the right knee to be able to cope with the slopes? And what's this slope technique like, it would prompt me to kind of think about some of the skills and make sure that they're good enough for him to be able to do what he needed to do.” (C1)

In summary, the interviewees had different clinical opinions on the participants' data, highlighting the need for an objective system that can support their clinical decision making. The different assessments of the system data gave further arguments for normative data to set benchmarks for each K level to take some bias out of their decision making. The differences highlighted between the clinical assessments and the system data emphasises the subjective nature of the clinical assessment and how patients can have different views on their own activity levels compared to what they actually achieve.

Theme 5: Areas for improvement

There were a couple of negative points that the interviewees made about the data presented, that were in relation to lack of clarity regarding some of the definitions, and also the extent to which activity data are separated in the system's output, both of which indicated areas of improvement for future work.

Lack of clarity

The definitions of uneven and unstable terrain were highlighted by three of the interviewees as areas of uncertainty within the system's data:

"The difference between uneven terrain or unstable terrain, and that, I don't know if I'd know what the difference was between that." (C1)

"I didn't really follow what you, how you differentiate them." (C2)

"What's the difference then, between uneven and unstable?" (C4)

No definitions were given in the report regarding this, so this is something that should be added in future.

Extent to which activity data are separated.

Two of the interviewees would have preferred if grass, uneven and unstable data were combined:

"You could combine all of your grass, uneven terrain and unstable terrain, into uneven terrain, and you could put it all in together." (C1)

"I'm just wondering if there's a need for it to be broken down into that detail, or if you can put them together." (C4)

C1 gave a justification for this:

"There's nothing in the K levels that differentiates the type of uneven surface. It just kind of talks about uneven surfaces." (C1)

In contradiction to this, interviewee C2 preferred them to be separate:

"I prefer them separate." (C2)

Justifying this with:

"The better you break it down, as long as people understand what the differences are, I think you have a better chance of being able to get the best prescription" (C2)

And they gave an example to further this argument:

“If you have someone, for example, who lives in Brighton and is down at the beach all the time, then they're going to have very different requirements than someone who lives in Sheffield and is living in the city centre.” (C2)

In summary, while clinicians were generally positive about the system, they identified several areas where improvements could enhance its usability and clinical relevance. In addition to desiring load measurement and wear and activity times, discussed under output data of the system, definitions need to be added to the system’s data, especially for uneven and unstable terrain. Furthermore, there were contrasting thoughts on whether the data needs to be split up into as may categories as it currently has been, and perhaps an option to combine when creating output summaries may be the solution.

Discussion

The system developed for assessing real-world activity in lower-limb prosthesis users was generally well-received by clinicians, who found its output to be both clinically relevant and useful in supporting their K level assessments. The measures that have been produced by the system were all deemed to be clinically relevant, with only load through the prosthesis and wear or active time as additional measures suggested that would be desirable.

Notably, all the interviewees gave positive feedback on the summary of the participants activities they were provided with, saying that having the data presented in a simple way and easy to read was useful. There were contrasting opinions about the daily activity table, however, some saying that having the daily data would be helpful in seeing how activity changed over the data collection period and could explain some discrepancies between the clinical assessment and the system data, but the interviewees had generally negative views on how it was displayed in that table. One suggestion some of the interviewees had was to visualise the data in some way. A bell curve of cadences was given as one example.

All the interviewees thought the system was clinically relevant and when comparing the system data to the clinical assessment summaries they thought that the sensor data could have changed the K level for some of the participants that they otherwise would not have assigned to them. They all thought the data would be very useful to have to add to their clinical assessment, to justify decisions, prompt in-depth questions on patients’ lives, and enable difficult conversations to be had objectively. The use of the data to prompt questions was a positive that the interviewees were very enthusiastic about, as it would allow them to get more information about their patients and, therefore, a greater understanding of their lives, which could help them ensure that their patients are getting the right prescription and support.

None of the interviewees thought that a system that needed to be charged every night would be an issue for lower limb prosthetic users. This is as long as the method of charging is easy and straight forward, and magnetic charging ports were suggested as a potential solution for this.

There was debate as to whether to have the activity data split as it currently is, or to combine uneven, grass and unstable terrain all together. The argument for combining these activities into one was that K level descriptions do not distinguish between different uneven terrains and that it would be easier to understand if they are combined. The argument for keeping them split is that it would give the clinicians more detail about their patients’ activities. Currently with the data split, the accuracies obtained for leave-some-out were 56.89% and 83.64% for cross-validation, with uneven, grass and unstable combined the accuracies increase to 65.70% for leave-some-out and 87.29% for

cross validation. Perhaps providing clinicians with an option to combine, for better accuracies and simpler interpretation, may be the solution.

Limitations of the collected data

The data collected would have been over different terrain and in different condition than that used to train the models. Hence it cannot be assumed that the same cross-validation accuracies were achieved. Due to this, the terrain classification and walking aid use were only an estimation. However, walking aid use was checked with the participants, and they all said they did not use a walking aid during the collection period which was similar to the model output.

Even with these limitations, the data were acceptable for the purpose of the study which had the aim to investigate clinicians' thoughts on the system and its output data, presented as examples of what the system is designed to produce. At this time, the data were not being used for any clinical decision making; further training data would be needed from a larger sample to further improve accuracies. Importantly, the interviewees were made aware of these limitations of the data before the interviews.

Conclusion

The system and the data that are produced were perceived as clinically relevant. Clinicians believed that the system's data on their patients' everyday life would enhance clinical decision making and improve outcomes for their patients, as well as help justify the prescription that they receive. Load through the prosthesis is a measure that clinicians do desire and which could also improve the classification accuracies, in particular for walking aid use. The summary of the prosthetic users' activities as it had been presented to clinicians was perceived as useful as is, but finding a better way to visualise the daily activities table data could make it easier for clinicians to understand.

References

1. Kallio H, Pietilä AM, Johnson M, Kangasniemi M. Systematic methodological review: developing a framework for a qualitative semi-structured interview guide. J Adv Nurs. 2016;72(12):2954–65.
2. Braun V, Clarke V. Using thematic analysis in psychology. Qualitative Research in Psychology. 2006;3(2):77–101.

Appendix

Interview Guide

Version (14-11-23)

Interviews are planned with all clinicians who assessed data from patients that took part in the real-world study to monitor lower limb prosthetic patient's activity levels using the data from the sensor system. These interviews will be conducted to explore the clinicians' views of the sensor system.

The Inclusion Criteria: 1) assessed patients' data from the sensor system

A general opening question may be "What are your thoughts on the sensor data?" which will be followed by a series of trigger questions throughout the interview, for example:

- Could you see any problems with the sensor system?
- Would the sensors affect your assessments?
- Do you find the data interesting?
- Would the data affect your activity level assessments?
- Does the data surprise you?
- Is the data useful?
- Is there any other data you would like?
- What are your thoughts on the presentation of the data?
- Is there any parts of the data you do not think are relevant?
- Are there any other improvement you would like?

For all interviewees, follow up questions may be asked such as:

- How did this affect you?
- How do you feel about this?
- Does this worry you?
- What do you think may help with this problem?
- Why do you think this is?
- What are your views on this?
