# Supplementary material for: Lower limb prosthetic users’ activity classification through real-world inertial sensing
Source: PLoS One. 2026 Oct 9;21(10):e0358882. doi: 10.1371/journal.pone.0358882 (PMC13652245; doi:10.1371/journal.pone.0358882)
Supplement: Supplementary file 2 [file pone.0358882.s002.pdf]

<u>Review – PLOS ONE (PONE-D-26-02026)</u>

Reviewer Comments

Thank you for considering me reviewing this manuscript “"Lower limb prosthetic users’ activity classification through real-world inertial sensing". Please see my comments below:

Abstract

1. The abstract frames the system as a crucial tool for improving prescriptions, yet LSO accuracies are modest (~56–62%). These risks overstating clinical applicability.
2. The difference between CV and LSO should be explained briefly; many readers equate CV with generalizable accuracy.
3. Phase-2 clinical evaluation (n=3) is very small; claims should be toned down.
4. Sensitivity/specificity or per-class performance would be more informative than overall accuracy.

Introduction

1. The introduction sometimes implies that objective data will directly improve prescription, but this link is not empirically tested.
2. Prior studies with similar LSO accuracies should be contextualized more critically.
3. The rationale for why shank placement should be sufficient could be elaborated biomechanically.

Methods

Participants

1. Only 2 females. Severe sex imbalance; discuss implications.
2. Broad mix of amputation levels; unclear whether models generalize equally.
3. Exclusion of high-activity users may bias K-level relevance.

Data collection

1. Terrain definitions rely partly on researcher judgement; reproducibility issue.
2. Number of strides per class is imbalanced.
3. Clarify whether walking speed was controlled or natural.

Signal processing

1. More justification is needed for the 5 Hz filter.
2. Person-specific normalization may reduce generalizability; please discuss.

Model development

1. Hyperparameter tuning process is not sufficiently described.
2. Clarify how overfitting was prevented.
3. Why was accuracy the only optimization metric?

Validation

1. Clarify: normality checks, multiple comparison correction, and effect sizes.

Results

1. Very large tables are difficult to interpret; consider summarizing key findings.
2. Confidence intervals might be clearer than SD.
3. Improvement after merging terrain classes appears post-hoc.
4. Per-participant variability would be useful.
5. n=3 users, 4 clinicians: Limited generalizability.
6. Experts reacted to reports, not real prescription decisions.

Discussion

1. What accuracy threshold clinicians would require,
2. How errors might change reimbursement or safety.

Conclusions

1. Currently it's too strong. The system shows potential, but further development and larger datasets are essential before clinical deployment.

Language and terminology

1. Optima vs optimal
2. Some figure cross-references appear broken.
3. Walking aid vs walking-aid

Recommendation

Major revision. High clinical relevance, but interpretation and reporting must better align with the achieved performance.
