# Supplementary material for: Lower limb prosthetic users’ activity classification through real-world inertial sensing
Source: PLoS One. 2026 Oct 9;21(10):e0358882. doi: 10.1371/journal.pone.0358882 (PMC13652245; doi:10.1371/journal.pone.0358882)
Supplement: Supplementary file 4 [file pone.0358882.s004.docx]

1. The values reported in the text are inconsistent with those shown in Figures 3 and 4. The authors should carefully verify all performance metrics and ensure consistency between the figures and the manuscript text. For example, in Line 508, according to Figure 3, 83.4% represents sensitivity, not specificity, whereas 50.8% represents precision, not sensitivity. The authors may find the following references useful for the appropriate calculation, application, and interpretation of these performance metrics: 10.1016/j.ipm.2009.03.002 and <https://doi.org/10.3390/bioengineering11121250>.

**Response**: We thank the reviewer for identifying this inaccuracy in our reporting. Precision, specificity and sensitivity is now correctly reported throughout the manuscript (Discussion lines 509, 510, 512, 515, 516, 528, 529, 530). We also added a sentence in the methods to state how these were calculated, Methods lines 334-338 “Per-class performance is reported via confusion matrix for the final model, with precision, sensitivity and specificity reported. For each class, sensitivity was calculated as TP/(TP+FN), precision as TP/(TP+FP), and specificity as TN/(TN+FP), where TP, TN, FP and FN denote true positives, true negatives, false positives and false negatives, respectively; metrics were calculated using a one-versus-rest approach”.

2. The authors should clarify how the accuracy values reported in Lines 424–425 were calculated.

**Response**: We thank the reviewer for identifying that the calculation and aggregation of the reported accuracy values was insufficiently described. We have revised the Methods and Results to clarify that accuracy was calculated at the stride-window level as the proportion of held-out windows correctly classified (Methods line 332-333 “Accuracy was calculated for each validation test set as the proportion of held-out stride windows correctly classified”). We now specify how mean accuracy, SD and 95% confidence intervals were calculated across validation iterations (Methods line 342-345 “The mean accuracy across the five test folds/groups are reported for both LSO and CV, with standard deviation (SD) calculated across fold/group accuracies and 95% confidence intervals (CI) calculated using the Student’s t distribution ($\pm t_{0.975,4}\times SD/\sqrt{5}$)”). The reported values have been revised to add clarity to this (results lines 425-428 “the re-optimised model achieved a mean LSO accuracy across held-out participant groups of **56.89% (****SD 5.5%; 95% CI 50.1–63.7%)** and a mean CV accuracy of **83.64% (SD 0.8%; 95% CI 82.6–84.7%)**” and lines 469-470 “and achieved a mean LSO accuracy across held-out participant groups of **61.56% (SD 16.1%; 95% CI 45.4-77.7%)** and a mean CV accuracy of **82.90% (SD 2.1%; 95% CI 80.8-85.0%)**”).

3. Figure 2 has no panel key. Please add a key mapping each panel to its terrain type.

**Response**: We thank the reviewer for identifying that Figure 2 does not have a panel key. A,B,C,D added to figure and key added to figure legend, Methods tine 274-275 “A) Uneven ground (cobble stones), B) Grass, C) Unstable gravel, D) Stairs.”.

4. The authors should conduct a thorough typographical and grammatical proofread of the manuscript. Examples include “prothesis” in Line 220 and “recorded” in Line 255.

**Response**: We thank the reviewer for identifying the errors in the manuscript. The manuscript has now been checked with mistakes corrected throughout the manuscript.
