# Supplementary material for: Lower limb prosthetic users’ activity classification through real-world inertial sensing
Source: PLoS One. 2026 Oct 9;21(10):e0358882. doi: 10.1371/journal.pone.0358882 (PMC13652245; doi:10.1371/journal.pone.0358882)
Supplement: Peer review history

# Peer review: Decision Letter 0

**DOI:** 10.1371/journal.pone.0358882.r001

**Authors:** Gary Guerra

24 Feb 2026

Dear Dr. Wassall,

Thank you for submitting your manuscript to PLOS ONE. After careful consideration, we feel that it has merit but does not fully meet PLOS ONE’s publication criteria as it currently stands. Therefore, we invite you to submit a revised version of the manuscript that addresses the points raised during the review process.

Please submit your revised manuscript by Apr 10 2026 11:59PM. If you will need more time than this to complete your revisions, please reply to this message or contact the journal office at plosone@plos.org. When you're ready to submit your revision, log on to https://www.editorialmanager.com/pone/ and select the 'Submissions Needing Revision' folder to locate your manuscript file.

- A letter that responds to each point raised by the academic editor and reviewer(s). You should upload this letter as a separate file labeled 'Response to Reviewers'.
- A marked-up copy of your manuscript that highlights changes made to the original version. You should upload this as a separate file labeled 'Revised Manuscript with Track Changes'.
- An unmarked version of your revised paper without tracked changes. You should upload this as a separate file labeled 'Manuscript'.

If you would like to make changes to your financial disclosure, please include your updated statement in your cover letter. Guidelines for resubmitting your figure files are available below the reviewer comments at the end of this letter.

If applicable, we recommend that you deposit your laboratory protocols in protocols.io to enhance the reproducibility of your results. Protocols.io assigns your protocol its own identifier (DOI) so that it can be cited independently in the future. For instructions see: https://journals.plos.org/plosone/s/submission-guidelines#loc-laboratory-protocols. Additionally, PLOS ONE offers an option for publishing peer-reviewed Lab Protocol articles, which describe protocols hosted on protocols.io. Read more information on sharing protocols at https://plos.org/protocols?utm_medium=editorial-email&utm_source=authorletters&utm_campaign=protocols.

We look forward to receiving your revised manuscript.

Kind regards,

Gary Guerra, Ph.D.

Academic Editor

PLOS One

Journal Requirements:

When submitting your revision, we need you to address these additional requirements.

1. Please ensure that your manuscript meets PLOS ONE's style requirements, including those for file naming. The PLOS ONE style templates can be found at

https://journals.plos.org/plosone/s/file?id=wjVg/PLOSOne_formatting_sample_main_body.pdf and

https://journals.plos.org/plosone/s/file?id=ba62/PLOSOne_formatting_sample_title_authors_affiliations.pdf

2. Please note that PLOS One has specific guidelines on code sharing for submissions in which author-generated code underpins the findings in the manuscript. In these cases, we expect all author-generated code to be made available without restrictions upon publication of the work. Please review our guidelines at https://journals.plos.org/plosone/s/materials-and-software-sharing#loc-sharing-code and ensure that your code is shared in a way that follows best practice and facilitates reproducibility and reuse.

3. Thank you for uploading your study's underlying data set. Unfortunately, the repository you have noted in your Data Availability statement does not qualify as an acceptable data repository according to PLOS's standards.

At this time, please upload the minimal data set necessary to replicate your study's findings to a stable, public repository (such as figshare or Dryad) and provide us with the relevant URLs, DOIs, or accession numbers that may be used to access these data. For a list of recommended repositories and additional information on PLOS standards for data deposition, please see https://journals.plos.org/plosone/s/recommended-repositories.

4. We note that there is identifying data in the Supporting Information file “S1 File.pdf”. Due to the inclusion of these potentially identifying data, we have removed this file from your file inventory. Prior to sharing human research participant data, authors should consult with an ethics committee to ensure data are shared in accordance with participant consent and all applicable local laws.

Data sharing should never compromise participant privacy. It is therefore not appropriate to publicly share personally identifiable data on human research participants. The following are examples of data that should not be shared:

-Name, initials, physical address

-Ages more specific than whole numbers

-Internet protocol (IP) address

-Specific dates (birth dates, death dates, examination dates, etc.)

-Contact information such as phone number or email address

-Location data

-ID numbers that seem specific (long numbers, include initials, titled “Hospital ID”) rather than random (small numbers in numerical order)

Data that are not directly identifying may also be inappropriate to share, as in combination they can become identifying. For example, data collected from a small group of participants, vulnerable populations, or private groups should not be shared if they involve indirect identifiers (such as sex, ethnicity, location, etc.) that may risk the identification of study participants.

Additional guidance on preparing raw data for publication can be found in our Data Policy (https://journals.plos.org/plosone/s/data-availability#loc-human-research-participant-data-and-other-sensitive-data) and in the following article: http://www.bmj.com/content/340/bmj.c181.long.

Please remove or anonymize all personal information (<specific identifying information in file to be removed>), ensure that the data shared are in accordance with participant consent, and re-upload a fully anonymized data set. Please note that spreadsheet columns with personal information must be removed and not hidden as all hidden columns will appear in the published file.

5. We note that this data set consists of interview transcripts. Can you please confirm that all participants gave consent for interview transcript to be published?

If they DID provide consent for these transcripts to be published, please also confirm that the transcripts do not contain any potentially identifying information (or let us know if the participants consented to having their personal details published and made publicly available). We consider the following details to be identifying information:

- Names, nicknames, and initials

- Age more specific than round numbers

- GPS coordinates, physical addresses, IP addresses, email addresses

- Information in small sample sizes (e.g. 40 students from X class in X year at X university)

- Specific dates (e.g. visit dates, interview dates)

- ID numbers

Or, if the participants DID NOT provide consent for these transcripts to be published:

- Provide a de-identified version of the data or excerpts of interview responses

- Provide information regarding how these transcripts can be accessed by researchers who meet the criteria for access to confidential data, including:

a) the grounds for restriction

b) the name of the ethics committee, Institutional Review Board, or third-party organization that is imposing sharing restrictions on the data

c) a non-author, institutional point of contact that is able to field data access queries, in the interest of maintaining long-term data accessibility.

d) Any relevant data set names, URLs, DOIs, etc. that an independent researcher would need in order to request your minimal data set.

For further information on sharing data that contains sensitive participant information, please see: https://journals.plos.org/plosone/s/data-availability#loc-human-research-participant-data-and-other-sensitive-data

If there are ethical, legal, or third-party restrictions upon your dataset, you must provide all of the following details (https://journals.plos.org/plosone/s/data-availability#loc-acceptable-data-access-restrictions):

a. A complete description of the dataset

b. The nature of the restrictions upon the data (ethical, legal, or owned by a third party) and the reasoning behind them

c. The full name of the body imposing the restrictions upon your dataset (ethics committee, institution, data access committee, etc)

d. If the data are owned by a third party, confirmation of whether the authors received any special privileges in accessing the data that other researchers would not have

e. Direct, non-author contact information (preferably email) for the body imposing the restrictions upon the data, to which data access requests can be sent

6. If the reviewer comments include a recommendation to cite specific previously published works, please review and evaluate these publications to determine whether they are relevant and should be cited. There is no requirement to cite these works unless the editor has indicated otherwise.

[Note: HTML markup is below. Please do not edit.]

Reviewers' comments:

Reviewer's Responses to Questions

**Comments to the Author**

1. Is the manuscript technically sound, and do the data support the conclusions?

Reviewer #1: Partly

Reviewer #2: Yes

Reviewer #3: Yes

**********

2. Has the statistical analysis been performed appropriately and rigorously?

Reviewer #1: No

Reviewer #2: N/A

Reviewer #3: Yes

**********

3. Have the authors made all data underlying the findings in their manuscript fully available?

The PLOS Data policy

Reviewer #1: Yes

Reviewer #2: Yes

Reviewer #3: Yes

**********

4. Is the manuscript presented in an intelligible fashion and written in standard English?

Reviewer #1: Yes

Reviewer #2: Yes

Reviewer #3: Yes

**********

Reviewer #1: A. General comments

This manuscript addresses a clinically important and timely problem: the subjectivity inherent in K-level assignment for lower-limb prosthesis users. The authors propose an IMU-based machine-learning framework for real-world classification of terrain and walking-aid use, complemented by a small clinical evaluation. Real-world, multi-terrain data were collected from an amputee cohort (n = 20). Overall, the motivation is well-founded, the topic is relevant to clinical practice, and the dataset represents a valuable contribution.

B. Major comments

1. Some wording in the abstract, for example, “significant shift” (line 35) and “crucial, evidence-based” (lines 36-37), appears overstated given the evaluation design and modest performance. The authors should temper these claims and ensure that similarly strong language is appropriately qualified throughout the manuscript. In addition, given the results, the work is better framed as a feasibility and methods paper, not as a clinically ready tool.

2. The introduction interweaves clinical motivation, technical limitations, and prior qualitative findings, but does not adequately synthesize these elements into a clearly articulated research question. As written, repeated statements indicating “no conclusive evidence” read as a descriptive list rather than a coherent, focused knowledge gap. Although five objectives are presented, explicit, testable hypotheses are not clearly defined. The authors should restructure this section to more clearly articulate the central research gap and align the objectives with corresponding hypotheses.

3. Additional detail on the terrain conditions is needed to improve clarity and reproducibility. Including representative photographs for each terrain is strongly recommended.

4. It is unclear whether terrain and walking-aid conditions were randomized. The authors should clarify this in the methods.

5. The manuscript states that IMU data were filtered with a 5 Hz-pass Butterworth filter. This is commonly low for gait classification and may remove meaningful gait dynamics. The authors should justify why 5 Hz was used. Was this applied to all IMU channels?

6. IMU features are derived from three axes (X, Y, Z), but it is unclear how these components were used. The authors should specify how multi-axis data were incorporated into the features and define how each IMU axis maps to anatomical directions.

7. The authors should justify the inclusion of each model parameter. For example, why IMU placement on the trunk is expected to provide information distinct from thigh placement, and why magnetic field strength is hypothesized to contribute to the model.

8. Model hyperparameters and optimization process should be specified.

9. The results of the normality tests underlying the ANOVA are not reported.

10. What is the dependent variable in ANOVA? The authors should also report F-values and effect sizes.

11. Figures 2 and 3 show heavy misclassification. It is recommended to provide per-class metrics in the main text rather than just matrices.

12. Some interview methodology details are missing, such as the interview guide, coding procedure, and inter-rater reliability.

13. The manuscript states that clinicians reviewed the IMU-derived reports, but there is no quantitative comparison to clinician K-level ratings. Did clinicians change their K-level assessment? Did IMU data increase confidence?

14. LSO accuracies remain low, barely exceeding baselines. While CV is higher, the gap suggests overfitting, potentially due to inter-subject variability in prosthetics or terrain definitions. Subgroup analyses are recommended to potentially reveal biases, e.g., do higher-level amputees show different patterns? The authors should also critically discuss why the model generalizes poorly to new individuals and how this might impact clinical adoption.

15. While 20 participants are reasonable for an initial ML study, the gender balance is heavily skewed. The authors should discuss how this might limit the generalizability of the findings to female prosthetic users.

16. The literature review could be strengthened by adding more key points, for example, recent prosthetic-specific IMU classification studies and terrain classification using deep learning architectures.

C. Minor comments

1. Clarity would be improved by explicitly specifying the final models in the abstract.

2. Were there any exclusive criteria applied?

3. Key demographic characteristics are missing, including time since amputation and current K-level.

4. “Other shank” (line 149) could be renamed, for example, “intact limb shank” for clarity.

5. “Unstable gravel” (line 157) should be defined more precisely.

6. Did all participants attempt all terrains? Any missing data?

7. The type of walking aid is not reported.

8. There is no description of IMU calibration.

Reviewer #2: Review – PLOS ONE (PONE-D-26-02026)

Reviewer Comments

Thank you for considering me reviewing this manuscript “"Lower limb prosthetic users’ activity classification through real-world inertial sensing". Please see my comments below:

Abstract

1. The abstract frames the system as a crucial tool for improving prescriptions, yet LSO accuracies are modest (~56–62%). These risks overstating clinical applicability.

2. The difference between CV and LSO should be explained briefly; many readers equate CV with generalizable accuracy.

3. Phase-2 clinical evaluation (n=3) is very small; claims should be toned down.

4. Sensitivity/specificity or per-class performance would be more informative than overall accuracy.

Introduction

1. The introduction sometimes implies that objective data will directly improve prescription, but this link is not empirically tested.

2. Prior studies with similar LSO accuracies should be contextualized more critically.

3. The rationale for why shank placement should be sufficient could be elaborated biomechanically.

Methods

Participants

1. Only 2 females. Severe sex imbalance; discuss implications.

2. Broad mix of amputation levels; unclear whether models generalize equally.

3. Exclusion of high-activity users may bias K-level relevance.

Data collection

1. Terrain definitions rely partly on researcher judgement; reproducibility issue.

2. Number of strides per class is imbalanced.

3. Clarify whether walking speed was controlled or natural.

Signal processing

1. More justification is needed for the 5 Hz filter.

2. Person-specific normalization may reduce generalizability; please discuss.

Model development

1. Hyperparameter tuning process is not sufficiently described.

2. Clarify how overfitting was prevented.

3. Why was accuracy the only optimization metric?

Validation

1. Clarify: normality checks, multiple comparison correction, and effect sizes.

Results

1. Very large tables are difficult to interpret; consider summarizing key findings.

2. Confidence intervals might be clearer than SD.

3. Improvement after merging terrain classes appears post-hoc.

4. Per-participant variability would be useful.

5. n=3 users, 4 clinicians: Limited generalizability.

6. Experts reacted to reports, not real prescription decisions.

Discussion

1. What accuracy threshold clinicians would require,

2. How errors might change reimbursement or safety.

Conclusions

1. Currently it’s too strong. The system shows potential, but further development and larger datasets are essential before clinical deployment.

Language and terminology

1. Optima vs optimal

2. Some figure cross-references appear broken.

3. Walking aid vs walking-aid

Recommendation

Major revision. High clinical relevance, but interpretation and reporting must better align with the achieved performance.

Reviewer #3: This work has some clinical relevance which directly targets a known problem (subjective/variable K-level assessment leading to poor prescriptions) with ecologically valid data collection (outdoor/real terrains, walking aid variations, researcher-present but aiming for free-living extension). This work also includes clinician input and addresses key variables (step count, cadence/variability, terrain, aid use).

Limited details on feature engineering (exact time/frequency-domain stats per window), normalization/filtering impacts (5 Hz low-pass Butterworth), outlier/missing data handling.

Appears to be no external hold-out validation Individual datapoint windows not fully tested with CV due to leakage risk. No test-retest reliability for stride detection/cadence in this cohort or IMU attachment repeatability on prosthesis pylon.

Stride labeling based on foot-contact terrain without analysis of swing/stance contributions or optimal labeling point

It might help to refine terrain definitions (e.g., objective slope/roughness metrics, ankle kinematics as alternative); explicitly label/handle transitions; analyze feature importance. Or at least explain them in a bit more detail for readers.

What were the exact features extracted per window (e.g., mean, SD, RMS, frequency stats)? How were hyperparameters tuned, and was there risk of overfitting in the step-wise optimization?

How were strides precisely labeled for terrain (e.g., heel-strike foot placement)? Were transition strides identified/excluded? Was stride detection validated on this dataset?

Overall, this is an ambitious, clinically motivated study with strong real-world data collection and open data practices.

**********

what does this mean?). If published, this will include your full peer review and any attached files.

If you choose “no”, your identity will remain anonymous but your review may still be made public.

**Do you want your identity to be public for this peer review?** For information about this choice, including consent withdrawal, please see our Privacy Policy

Reviewer #1: No

Reviewer #2: **Yes:**  Dr. Huthaifa Atallah

Reviewer #3: No

**********

[NOTE: If reviewer comments were submitted as an attachment file, they will be attached to this email and accessible via the submission site. Please log into your account, locate the manuscript record, and check for the action link "View Attachments". If this link does not appear, there are no attachment files.]

To ensure your figures meet our technical requirements, please review our figure guidelines: https://journals.plos.org/plosone/s/figures

You may also use PLOS’s free figure tool, NAAS, to help you prepare publication quality figures: https://journals.plos.org/plosone/s/figures#loc-tools-for-figure-preparation.

NAAS will assess whether your figures meet our technical requirements by comparing each figure against our figure specifications.

Attachment Submitted filename: renamed_1dc7b.pdf

# Author comment: Author response to Decision Letter 1

**DOI:** 10.1371/journal.pone.0358882.r002

15 Jul 2026

Reviewer 1

1. Some wording in the abstract, for example, “significant shift” (line 35) and “crucial, evidence-based” (lines 36-37), appears overstated given the evaluation design and modest performance. The authors should temper these claims and ensure that similarly strong language is appropriately qualified throughout the manuscript. In addition, given the results, the work is better framed as a feasibility and methods paper, not as a clinically ready tool.

Response: We agree and have tempered the language throughout the manuscript to better align with the observed performance and feasibility‑focus of the study. The abstract and Conclusion now describe the system as an exploratory feasibility and methods approach rather than a clinically ready tool. Abstract lines 38, 40, 43-47, Conclusion lines 601, 608, 611-612.

2. The introduction interweaves clinical motivation, technical limitations, and prior qualitative findings, but does not adequately synthesize these elements into a clearly articulated research question. As written, repeated statements indicating “no conclusive evidence” read as a descriptive list rather than a coherent, focused knowledge gap. Although five objectives are presented, explicit, testable hypotheses are not clearly defined. The authors should restructure this section to more clearly articulate the central research gap and align the objectives with corresponding hypotheses.

Response: we agree and now comment that it remains unclear which combination of sensor placement, window definition, sampling density and IMU features offers the best trade off between classification performance and clinical feasibility and identify this as the central research gap our study addresses. We have also added hypothesis for each objective. Introduction lines 141-148, 153-175

3. Additional detail on the terrain conditions is needed to improve clarity and reproducibility. Including representative photographs for each terrain is strongly recommended.

Response: We thank the reviewer for this helpful suggestion. We have expanded the description of each terrain in the Methods to specify how flat, ramp, stairs, uneven ground and unstable gravel were operationally defined, to improve clarity and reproducibility. In addition, we have added representative photographs illustrating each terrain type as Fig 2. Methods lines 232-235, 244

4. It is unclear whether terrain and walking-aid conditions were randomized. The authors should clarify this in the methods.

Response: We agree that this should be clarified. We have added a statement to the Methods specifying that the order of terrain conditions and the presence or absence of a walking aid were randomised for each participant to minimise order effects. methods lines 230-231

5. The manuscript states that IMU data were filtered with a 5 Hz-pass Butterworth filter. This is commonly low for gait classification and may remove meaningful gait dynamics. The authors should justify why 5 Hz was used. Was this applied to all IMU channels?

Response: We agree that this required clarification. In the revised Methods we now state that a 5 Hz low‑pass Butterworth filter was applied to all IMU channels (acceleration, free‑acceleration, angular velocity and magnetic field). The 5 Hz cut‑off was chosen based on a frequency analysis of resultant acceleration from a representative subset of strides across terrains and walking‑aid conditions, which showed that the dominant gait‑related power was concentrated below approximately 4 Hz, so the filter was expected to preserve relevant gait dynamics while attenuating higher‑frequency noise. See Methods lines 255-258.

6. IMU features are derived from three axes (X, Y, Z), but it is unclear how these components were used. The authors should specify how multi-axis data were incorporated into the features and define how each IMU axis maps to anatomical directions.

Response: We agree and have clarified both the axis definitions and how multi‑axis data were used. In the revised Methods we now state that the IMUs were mounted so that the local X, Y and Z axes were approximately aligned with the segment’s vertical (longitudinal), medio‑lateral and antero‑posterior directions, respectively, for all sensor locations. We also specify that for each channel the tri‑axial signals (X, Y, Z) were included separately in the models, and that we additionally computed the resultant magnitude for acceleration and free‑acceleration as axis‑invariant features. See Methods lines 213-223

7. The authors should justify the inclusion of each model parameter. For example, why IMU placement on the trunk is expected to provide information distinct from thigh placement, and why magnetic field strength is hypothesized to contribute to the model.

Response: We agree and have expanded the Introduction to clarify the rationale for each model parameter. We now explain that shank, thigh and trunk placements are expected to capture partly distinct information because segment kinematics differ across the lower limb and trunk when negotiating different terrains and using a walking aid. In addition, we justify inclusion of magnetic field strength by noting that it provides complementary information on sensor heading and orientation relative to the environment, which can differ between terrains such as slopes versus level ground and between stair ascent and descent. Introduction lines 116-124, 141-148, Methods line 217-221

8. Model hyperparameters and optimization process should be specified.

Response: We agree and have expanded the Methods to describe the hyperparameter optimisation procedure. For each algorithm, an automatic Bayesian hyperparameter optimiser was run once on a representative subset of the data to obtain default settings, using held‑out validation loss to select the best combination. The resulting optimal values were very similar across window types, and where paired optima differed by less than 5% of the larger value we used their mean as the final setting. See Methods lines 307-314

9. The results of the normality tests underlying the ANOVA are not reported.

Response: We agree that the normality checks should be described more clearly. In the revised Methods, we now state that for each set of model accuracies we assessed normality using the Shapiro–Wilk test. When the normality assumption was not violated (p≥0.05), we applied ANOVA with Tukey’s HSD post‑hoc tests; when it was violated (p<0.05), we instead used Kruskal–Wallis tests with Dunn’s post‑hoc comparisons. We have not listed every Shapiro–Wilk statistic in the Results because this would add substantial length without changing interpretation; instead, the revised Methods define the decision rule and the Results report the corresponding test statistics for each comparison. Methods lines 328-337

10. What is the dependent variable in ANOVA? The authors should also report F-values and effect sizes.

Response: We agree that this required clarification. In the revised Methods we now state explicitly that the dependent variable in all ANOVA/Kruskal–Wallis tests was model classification accuracy, with factors such as algorithm, sensor placement, window type and sampling rate as independent variables. We have also added the corresponding F‑values (or chi‑squared statistics for non‑parametric tests) and effect sizes: partial eta‑squared for omnibus ANOVA effects and Cohen’s d for key pairwise post‑hoc comparisons. methods line 328-337 results lines 368-370, 373, 379-382, 383-389, 391-394, 408-409, 419-420, 425-426, 429-430, 438-440, 450-451.

11. Figures 2 and 3 show heavy misclassification. It is recommended to provide per-class metrics in the main text rather than just matrices.

Response: We agree that per‑class metrics improve interpretability. In the revised Results, we now report per‑class sensitivities for the models in the main text, alongside the confusion matrices, so that readers can see which classes are most frequently misclassified. results lines 489, 494-497, 506

12. Some interview methodology details are missing, such as the interview guide, coding procedure, and inter-rater reliability.

Response: We agree and coding procedure and interview guide added to supporting document. Methods lines 353-354

13. The manuscript states that clinicians reviewed the IMU-derived reports, but there is no quantitative comparison to clinician K-level ratings. Did clinicians change their K-level assessment? Did IMU data increase confidence?

Response: We agree that a quantitative comparison with clinician K‑level ratings would be valuable. In this study, clinicians reviewed IMU‑derived reports and provided qualitative feedback, but they were not asked to assign or revise K‑levels, and we did not measure changes in K‑level decisions or rating confidence. We have clarified in the Results/Discussion that clinicians responded to the reports rather than making formal K‑level assessments, and that the present findings therefore cannot determine whether, or how, the system would change K‑level assignments or increase clinicians’ confidence. Results lines 582-588

14. LSO accuracies remain low, barely exceeding baselines. While CV is higher, the gap suggests overfitting, potentially due to inter-subject variability in prosthetics or terrain definitions. Subgroup analyses are recommended to potentially reveal biases, e.g., do higher-level amputees show different patterns? The authors should also critically discuss why the model generalizes poorly to new individuals and how this might impact clinical adoption.

Response: We agree that the gap between cross‑validation and LSO accuracies indicates limited generalisation to new individuals, likely due in part to inter‑subject variability in prostheses, gait patterns and terrain exposure. We have added a leave‑one‑participant‑out analysis and expanded the Discussion to examine participant‑level variability, potential biases and implications for clinical adoption. Results line 410-416, 452-457, discussion lines 497-500, 527-530

15. While 20 participants are reasonable for an initial ML study, the gender balance is heavily skewed. The authors should discuss how this might limit the generalizability of the findings to female prosthetic users.

Response: We agree that the skewed sex distribution is an important limitation. In the revised Methods we now compare our sample to UK lower‑limb prosthetic user demographics, noting that only 10% of participants were female versus approximately 30% nationally, and that this mismatch may limit the generalisability of our findings, particularly to female prosthetic users. Methods lines 197-204

16. The literature review could be strengthened by adding more key points, for example, recent prosthetic-specific IMU classification studies and terrain classification using deep learning architectures.

Response: We agree and have expanded the literature review to include additional prosthetic‑specific IMU classification studies and recent work on terrain classification. Introduction lines 85-95, 120-124, 127-131

C. Minor comments

1. Clarity would be improved by explicitly specifying the final models in the abstract.

Response: We agree and clarity of final model added to results. Results lines 407, 449

2. Were there any exclusive criteria applied?

Response: We agree and exclusion criteria added to methods. methods lines 192-194

3. Key demographic characteristics are missing, including time since amputation and current K-level.

Response: We agree and further explanation of demographic characteristics added to methods. We did not record K-level because many participants were unaware of their classification. Similarly, time since amputation was not recorded. Instead, we prioritised functional ability (e.g., the ability to independently traverse various terrains) as a more reliable and direct inclusion criterion for this study. We have added further explanation of these demographics to the Methods. See Methods lines 196-207

4. “Other shank” (line 149) could be renamed, for example, “intact limb shank” for clarity.

Response: We agree and “other shank” has been changed to “contralateral shank” throughout manuscript.

5. “Unstable gravel” (line 157) should be defined more precisely.

Response: We agree and explanation of gravel added to methods. methods lines 232-234

6. Did all participants attempt all terrains? Any missing data?

Response: We agree that this should be clarified. We have added Table 1, which summarises which terrain and walking‑aid conditions each participant completed and therefore makes clear that not all participants attempted every terrain. We also note in the Methods that these un-attempted conditions represent missing data for those terrain classes and explain that models were trained on the available strides only. See Methods lines 238-242.

7. The type of walking aid is not reported.

Response: Thank you for the suggestion and “The walking aid used in the study was a walking stick” has been added to methods. See Methods line 231.

8. There is no description of IMU calibration.

Response: We agree and IMU calibration explanation added to methods. See Methods lines 223-225.

Reviewer 2

Abstract

1. The abstract frames the system as a crucial tool for improving prescriptions, yet LSO accuracies are modest (~56–62%). These risks overstating clinical applicability.

Response: We agree and language toned down. Abstract lines 38-47

2. The difference between CV and LSO should be explained briefly; many readers equate CV with generalizable accuracy.

Response: sentence added to explain the accuracy difference. Abstract lines 31-35 “The difference between cross‑validation and leave‑some‑out accuracies indicates participant‑specific overfitting, so the leave-some-out accuracy is considered the current conservative estimate of performance on unseen users and the cross-validation accuracy an estimate on potential accuracy with a more robust dataset”

3. Phase-2 clinical evaluation (n=3) is very small; claims should be toned down.

Response: Language toned down. Abstract lines 38-47

4. Sensitivity/specificity or per-class performance would be more informative than overall accuracy.

Response: per-class sensitivity added to abstract. Abstract lines 29-31 “Per‑class sensitivities ranged from approximately 44–78% in leave‑some‑out analyses, with lower performance for ramp ascending and descending.”

Introduction

1. The introduction sometimes implies that objective data will directly improve prescription, but this link is not empirically tested.

Response: Clarification added to explain potential impact but current evidence is not explored. Introduction line 59-63 “One solution to improve the reliability of K level assessments could be the use of activity monitoring in the real world, to provide clinicians with objective every-day data on activities which could support clinicians in making more consistent K level assessments and justify component selection, but this relationship has not yet been empirically demonstrated.”

2. Prior studies with similar LSO accuracies should be contextualized more critically.

Response: Added analysis of previous study that used a LSO approach. Introduction lines 127-131 “The only previous study found that classified terrain for lower limb prosthetic users using a wearable sensor and assessed using a leave-one-out approach achieved a 56.68% mean accuracy, but this study had only recruited 4 prosthetic users and only classified between flat, stairs (ascending and descending) and slopes (ascending and descending)”.

3. The rationale for why shank placement should be sufficient could be elaborated biomechanically.

Response: rationale added to why it was believed shank IMU placement would produce different IMU signals. Introduction lines 116-120 “Reported differences in knee and ankle kinematics across varying terrains indicate that an IMU positioned on the shank is likely to produce distinguishable signals (44). Confirming this a single shank-mounted IMU can produce good terrain classification accuracies (0.90 F1-score) on able bodied individuals (35), but this has not been investigated on lower limb prosthetic users”

Methods

Participants

1. Only 2 females. Severe sex imbalance; discuss implications.

Response: explanation of demographics against recorded UK lower limb prosthetic user demographics added and statement about how this could affect the generalisations of findings. Methods lines 197-204 “The participant demographics did not perfectly match those of UK lower limb prosthetic users: 10% of participants were female versus approximately 30% nationally, and 85% were older than 54 years compared with around 70% in the UK population, however, the distribution of amputation levels was similar (transtibial 55% vs 54%, transfemoral 40% vs 37%, bilateral 5% vs 6%), reflecting representativeness of the data in that regard. For pragmatic reasons we did not sample to exactly mirror national demographics, which may limit generalisability of the findings, particularly with respect to sex‑specific and age‑related patterns of activity.”

2. Broad mix of amputation levels; unclear whether models generalize equally.

Response: The distribution of amputation level was broadly similar to UK service demographics, supporting relevance to the target population; however, the sample remains too small to conclude that model performance generalises equally across amputation levels.

A statement was also added to methods that states preliminary analysis that showed no significant difference to classification accuracy between amputation level. Methods line 301-306“Participants included transtibial, transfemoral and one bilateral amputee, and all models were trained on the combined dataset without stratification by amputation level. Analyses were carried out that did not show significant differences in classification accuracy between transtibial and transfemoral subgroups (6), however, the present study cannot exclude that performance varies by amputation level and this remains a limitation.”

3. Exclusion of high-activity users may bias K-level relevance.

Response: The research was aimed at K2/K3 users, which was identified in the pre-study clinical interviews. This context has now been added to the introduction and clarification added to inclusion and exclusion criteria. Introduction lines 78-79 “It was also discovered that the issues mainly occur in decisions between K2 and K3 users.” Methods lines 190-194 “As the issues with K level assessments predominantly occur with K2 and K3 users, we used the following exclusion criteria:, not regularly participating in active sport with their prosthesis (football, running, etc.) and not primarily using a wheelchair,; we note that this could limit the generalisability of the findings to K1 and K4 users.”

Data collection

1. Terrain definitions rely partly on researcher judgement; reproducibility issue.

Response: The Methods now define the terrain categories more precisely and explicitly note where researcher judgement was used, particularly for uneven ground. This is stated in Methods, lines 231-235. We also signpost that this subjectivity is considered in the Discussion, lines 531-542, as a potential contributor to misclassification.

2. Number of strides per class is imbalanced.

Response: clarification about decision on stride per class imbalance added to methods. Methods lines 246-251 “The number of recorded strides per terrain class was imbalanced, reflecting the practical difficulty of collecting data on different terrain. Class imbalance may have contributed to poorer performance for under-represented or more heterogeneous classes, but it was considered low for the data set due to the marked biomechanical differences in traversing the different terrains. In the discussion individual class imbalances are discussed”. (Discussion lines 488-508, 527-530)

3. Clarify whether walking speed was controlled or natural

Response: statement added to methods to explain that participants walked at self-selected walking speed. Methods lines 251-253 “Participants walked at their self-selected natural walking speed on each terrain, which increases ecological validity of the dataset.”

Signal processing

1. More justification is needed for the 5 Hz filter.

Response: clarification on why 5Hz was used for the filter was added to the methods. Methods lines 255-258“The cut‑off was selected based on a frequency analysis of resultant acceleration from a representative subset of strides across terrains and walking aid conditions, which showed the dominant gait‑related power below 4 Hz.”

2. Person-specific normalization may reduce generalizability; please discuss.

Response: clarification added to methods of why normalization method was chosen and reference to preliminary analysis which identified the best normalization methods. Methods 258-268 “A combination of per‑person normalised and non-normalised data were used in the models to stabilise training while preserving absolute between‑subject differences. Normalisation is known to improve optimisation and reduce sensitivity to scale, but purely person‑specific normalisation can attenuate clinically relevant between‑subject variation; including both representations was therefore chosen as a pragmatic compromise in this feasibility study (52, 53). To our knowledge, combining per person normalised and raw IMU channels within the same feature set has not been reported previously, but in preliminary analysis (6) this combination produced the most favourable balance of cross validation and leave some out accuracies, so it was adopted for the present models.

Model development

1. Hyperparameter tuning process is not sufficiently described.

Response: clarification on hyperparameter tuning added to methods. Methods lines 307-314 “For each algorithm a Bayesian hyperparameter optimiser was run to mitigate overfitting. The optimisation was run on a representative subset of the data to obtain reasonable default settings. The optimiser evaluated a loss function on held‑out data, seeking the hyperparameter combination that minimised this loss. This initial optimisation was performed once per algorithm using 100 Hz data with 0.2 s windows and stride‑based windows (100 datapoints per stride). The optimal values were very similar across window types, and the mean of these values where small differences (<5%) occurred were used.”

2. Clarify how overfitting was prevented.

Response: We did apply a Bayesian hyperparameter optimiser to mitigate overfitting, as detailed in the Methods. However, we acknowledge overfitting could still be a limitation with a statement added to the discussion. Sensitivity and specificity of final model presented via confusion matrix and per-participant performance added to results, which presents any overfitting that occurred. Discussion lines 527-530 “The cross‑validation accuracies being significantly higher than leave‑some‑out accuracies indicates participant‑specific overfitting could be present. No class‑weighted training or additional regularisation was applied to the models, which could be addressed in future work.”

3. Why was accuracy the only optimization metric?

Response: statement added to methods that states that the primary optimisation metric was overall accuracy because the target is correct class assignment across all categories and to be comparable to prior studies. Methods lines 320-323 “Overall percentage accuracy was used as the primary optimisation metric because the target application is multi‑class terrain and walking aid recognition, where correct class assignment across all categories is clinically most relevant and directly comparable with prior studies”.

Validation

1. Clarify: normality checks, multiple comparison correction, and effect sizes.

Response: statement added to methods to clarify normality checks, multiple comparison and effect size. Effect size results also added to results. Methods lines 328-337 “Before comparing model accuracies, normality was assessed for each set of values using the Shapiro–Wilk test. When all models for a given comparison (e.g. windowing method, IMU position, feature set) satisfied the normality assumption (ρ≥0.05), a one‑way ANOVA (with F-value (F), degrees of freedom and ρ-value (ρ) reported) was used with Tukey’s HSD post‑hoc tests. Kruskal-Wallis (with Chi-square (X2), degrees of freedom and ρ values (ρ) reported) with Dunn’s post-hoc test was used for non-parametric data. Significance was accepted as ρ values lower than 0.05. For significant ANOVA effects we report partial eta‑squared as an effect size; for key pairwise post‑hoc contrasts we additionally report Cohen’s d.”

Results

1. Very large tables are difficult to interpret; consider summarizing key findings.

Response: The full table has been retained because it provides transparency across models and processing choices, but the Results text has been revised so that readers are not required to interpret the table unaided. A summary sentence now introduces the table and states that the principal findings are summarised in the following results paragraphs. Clarity added to Results lines 357-359 “The mean accuracies for all models tested for terrain classification and walking aid recognition are presented in Table 2, but the main findings are summarised in the text that follows.”

2. Confidence intervals might be clearer than SD.

Response: confidence intervals added for key results. Results lines 408-409,450-451, discussion lines 504-505.

3. Improvement after merging terrain classes appears post-hoc.

Response: clarification added to results. Discussion lines 500-505 “The clinical experts in Phase 2 suggested combining grass, uneven and unstable terrain categories into one uneven terrain category. In response to their comment, a RF model with these terrain categories was created for supplementary data analysis which then produced an accuracy increase to 65.70% (SD 6.6%; 95% CI 47.3–84.1%) for LSO and to 85.65% (SD 0.79%; 95% CI 83.4-87.9%) for CV.”

4. Per-participant variability would be useful.

Response: per-participant performance added to results. Results lines 410-416 “To investigate participant variability leave-one-participant-out (LOPO) analysis was run using the final RF model, Table 3. Mean accuracies between amputation levels were similar (TT 51.8±14.6%, TF 50.4±20.0%), and Welch’s t‑test showed no significant difference between groups (p=0.86), suggesting that amputation level did not influence classification accuracy.” Table 3 and lines 452-457 “LOPO walking aid recognition accuracies showed a large inter participant variability, Table 3, but amputation level did not affect model accuracies (TT 56.6±19.0%, TF 53.2±21.4%; Welch’s t‑test p=0.71). The proportion of steps performed with a walking aid was also not significantly associated with walking aid recognition accuracy (Pearson r=−0.10, p=0.65; Spearman ρ=−0.29, p=0.20), suggesting that uneven exposure to aided steps did not systematically bias performance.”

5. n=3 users, 4 clinicians: Limited generalizability. 6. Experts reacted to reports, not real prescription decisions.

Response: paragraph added to discussion that explains the limitation of the study and that the results should not be generalised to how the system could affect clinical outcomes. Discussion lines 582-588 “Phase‑2 of the study only involved 4 clinical experts analysing reports on 3 prosthetic users. The small participant population should be considered and caution taken when generalising the findings. Moreover, clinicians responded to simulated reports rather than using the system to make assessments decisions, so we cannot determine whether, or how, the information would actually change K‑level assignments or component selection in routine practice.”

Discussion

1. What accuracy threshold clinicians would require,

2. How errors might change reimbursement or safety.

Response: paragraph added to discussion that comments on the limitation that the study did not establish acceptable accuracy thresholds or how errors could affect clinical decision making. Discussion lines 589-596 “This research did not establish accuracy thresholds clinicians would consider sufficient for K‑level assessments or prescription decisions. How specific errors might influence clinical decisions was also not investigated. In Phase 2, clinicians commented that inaccuracies could be alleviated by combining system outcomes with current assessment methods rather than replacing these. Future work should include prospective studies in which clinicians use system outputs in real prescription workflows, to determine acceptable accuracy levels and identify which types of errors are clinically tolerable, also under consideration of risks to safety.”

Conclusions

1. Currently it’s too strong. The system shows potential, but further development and larger datasets are essential before clinical deployment.

Response: language toned down in conclusion to better reflect the outcomes of the study and limitations. Conclusion line 601, 608-612.

Reviewer 3

Limited details on feature engineering (exact time/frequency-domain stats per window), normalization/filtering impacts (5 Hz low-pass Butterworth), outlier/missing data handling.

Response: We have expanded the Signal processing and Feature description subsections to clarify these points. We clarify that all IMU channels (acceleration, angular velocity, magnetic field and free acceleration) were filtered using a 5 Hz low pass Butterworth filter and justify this cut off based on frequency analysis of representative strides across terrains. See Methods line 213-225, 255-264

Appears to be no external hold-out validation Individual datapoint windows not fully tested with CV due to leakage risk. No test-retest reliability for stride detection/cadence in this cohort or IMU attachment repeatability on prosthesis pylon.

Response: No external hold out validation was conducted instead, LSO analysis was conducted and further LOPO analysis has been added to results. These analyses validate the models ability to classify unseen participants. We did not perform a new test–retest reliability study for stride detection, cadence or IMU attachment repeatability on the prosthesis pylon in this cohort. Instead, we used a previously validated stride‑detection and cadence algorithm based on the prosthetic‑shank medio‑lateral angular velocity, which in earlier work with lower‑limb prosthesis users achieved high detection accuracy and precision, and we standardised IMU mounting on the prosthesis pylon. We now acknowledge in the Discussion that we did not quantify day‑to‑day variability in IMU placement or the test–retest reliability of derived measures in this sample, and identify this as an important area for future work. We intentionally did not apply cross‑validation to individual‑datapoint windows, because datapoints from the same stride would otherwise be split across training and validation sets, causing severe information leakage and artificially inflated accuracies. We have clarified this in the Methods. Methods lines 282-285, Results 410-416, 452-457 Discussion line 579-581

Stride labeling based on foot-contact terrain without analysis of swing/stance contributions or optimal labeling point.

Response: We thank the reviewer for this comment. In the revised Methods we now clarify that each stride was labelled according to the terrain under the prosthetic foot at initial contact (heel strike), so that the label reflects the terrain over which the majority of the stance phase occurs. We did not perform a separate analysis of swing‑ versus stance‑phase signal contributions or alternative labelling points (e.g. mid‑stance or mid‑swing). See Methods lines 294-297.

It might help to refine terrain definitions (e.g., objective slope/roughness metrics, ankle kinematics as alternative); explicitly label/handle transitions; analyse feature importance. Or at least explain them in a bit more detail for readers.

Response: In the Methods, we now more precisely define each terrain class as used in the study (flat ground, stairs ascending/descending, ramps steeper than 1:20, uneven hard surfaces such as cobblestones, and unstable gravel defined as a loose, small‑stone surface that visibly deformed underfoot with each step). We acknowledge that roughness was determined pragmatically by the experimenter rather than via objective surface metrics and now discuss this subjectivity as a potential contributor to misclassification in the Discussion. We also clarify that strides were labelled according to the terrain under the prosthetic foot at initial contact, and that transition strides were not treated differently. Feature importance was not conduced but in model analysis feature sets were analysed. Methods lines 231-238 294-297.

What were the exact features extracted per window (e.g., mean, SD, RMS, frequency stats)? How were hyperparameters tuned, and was there risk of overfitting in the step-wise optimization?

Response: features were not extracted from IMU signals apart from cadence and resultant accelerations. Hyperparameter tuning explanation has been added to methods. Bayesian hyperparameter optimisation was run not step-wise, this has now been explained in methods. Methods line 213-223, 307-314.

How were strides precisely labelled for terrain (e.g., heel-strike foot placement)? Were transition strides identified/excluded? Was stride detection validated on this dataset?

Response: We agree that these details should be made explicit and have clarified them in the Methods. Strides were detected using prosthetic‑shank medio‑lateral angular velocity, and each stride was labelled according to the terrain under the prosthetic foot at heel strike. Transition strides were not separately identified. See Methods lines 294-297.

Attachment Submitted filename: Response to Reviewers.docx

# Peer review: Decision Letter 1

**DOI:** 10.1371/journal.pone.0358882.r003

**Authors:** Gary Guerra

10 Aug 2026

Dear Dr. Wassall,

Thank you for submitting your manuscript to PLOS ONE. After careful consideration, we feel that it has merit but does not fully meet PLOS ONE’s publication criteria as it currently stands. Therefore, we invite you to submit a revised version of the manuscript that addresses the points raised during the review process.

Please submit your revised manuscript by Sep 24 2026 11:59PM. If you will need more time than this to complete your revisions, please reply to this message or contact the journal office at plosone@plos.org. When you're ready to submit your revision, log on to https://www.editorialmanager.com/pone/ and select the 'Submissions Needing Revision' folder to locate your manuscript file.

- A letter that responds to each point raised by the academic editor and reviewer(s). You should upload this letter as a separate file labeled 'Response to Reviewers'.
- A marked-up copy of your manuscript that highlights changes made to the original version. You should upload this as a separate file labeled 'Revised Manuscript with Track Changes'.
- An unmarked version of your revised paper without tracked changes. You should upload this as a separate file labeled 'Manuscript'.

If you would like to make changes to your financial disclosure, please include your updated statement in your cover letter. Guidelines for resubmitting your figure files are available below the reviewer comments at the end of this letter.

If applicable, we recommend that you deposit your laboratory protocols in protocols.io to enhance the reproducibility of your results. Protocols.io assigns your protocol its own identifier (DOI) so that it can be cited independently in the future. For instructions see: https://journals.plos.org/plosone/s/submission-guidelines#loc-laboratory-protocols. Additionally, PLOS ONE offers an option for publishing peer-reviewed Lab Protocol articles, which describe protocols hosted on protocols.io. Read more information on sharing protocols at https://plos.org/protocols?utm_medium=editorial-email&utm_source=authorletters&utm_campaign=protocols.

As the corresponding author, your ORCID iD is verified in the submission system and will appear in the published article. PLOS supports the use of ORCID, and we encourage all coauthors to register for an ORCID iD and use it as well. Please encourage your coauthors to verify their ORCID iD within the submission system before final acceptance, as unverified ORCID iDs will not appear in the published article. *Only* the individual author can complete the verification step; PLOS staff *cannot* verify ORCID iDs on behalf of authors.

We look forward to receiving your revised manuscript.

Kind regards,

Gary Guerra, Ph.D.

Academic Editor

PLOS One

Journal Requirements:

If the reviewer comments include a recommendation to cite specific previously published works, please review and evaluate these publications to determine whether they are relevant and should be cited. There is no requirement to cite these works unless the editor has indicated otherwise.

Please review your reference list to ensure that it is complete and correct. If you have cited papers that have been retracted, please include the rationale for doing so in the manuscript text, or remove these references and replace them with relevant current references. Any changes to the reference list should be mentioned in the rebuttal letter that accompanies your revised manuscript. If you need to cite a retracted article, indicate the article’s retracted status in the References list and also include a citation and full reference for the retraction notice.

[Note: HTML markup is below. Please do not edit.]

Reviewers' comments:

Reviewer's Responses to Questions

**Comments to the Author**

Reviewer #1: (No Response)

Reviewer #3: All comments have been addressed

**********

2. Is the manuscript technically sound, and do the data support the conclusions?

Reviewer #1: Partly

Reviewer #3: Yes

**********

3. Has the statistical analysis been performed appropriately and rigorously?

Reviewer #1: Yes

Reviewer #3: Yes

**********

4. Have the authors made all data underlying the findings in their manuscript fully available?

The PLOS Data policy

Reviewer #1: Yes

Reviewer #3: Yes

**********

5. Is the manuscript presented in an intelligible fashion and written in standard English?

Reviewer #1: No

Reviewer #3: Yes

**********

Reviewer #1: The authors have substantially revised the manuscript in response to the previous comments. However, several concerns still need to be addressed before the manuscript can be considered further.

1. The values reported in the text are inconsistent with those shown in Figures 3 and 4. The authors should carefully verify all performance metrics and ensure consistency between the figures and the manuscript text. For example, in Line 508, according to Figure 3, 83.4% represents sensitivity, not specificity, whereas 50.8% represents precision, not sensitivity. The authors may find the following references useful for the appropriate calculation, application, and interpretation of these performance metrics: 10.1016/j.ipm.2009.03.002 and https://doi.org/10.3390/bioengineering11121250.

2. The authors should clarify how the accuracy values reported in Lines 424–425 were calculated.

3. Figure 2 has no panel key. Please add a key mapping each panel to its terrain type.

4. The authors should conduct a thorough typographical and grammatical proofread of the manuscript. Examples include “prothesis” in Line 220 and “recorded” in Line 255.

Reviewer #3: The manuscript addresses an important and relevant topic and is well designed and clearly presented. The methods are appropriate, the analyses are sound, and the conclusions are supported by the data. I have no major concerns regarding the manuscript and recommend its acceptance for publication.

**********

what does this mean?). If published, this will include your full peer review and any attached files.

If you choose “no”, your identity will remain anonymous but your review may still be made public.

**Do you want your identity to be public for this peer review?** For information about this choice, including consent withdrawal, please see our Privacy Policy

Reviewer #1: No

Reviewer #3: No

**********

[NOTE: If reviewer comments were submitted as an attachment file, they will be attached to this email and accessible via the submission site. Please log into your account, locate the manuscript record, and check for the action link "View Attachments". If this link does not appear, there are no attachment files.]

To ensure your figures meet our technical requirements, please review our figure guidelines: https://journals.plos.org/plosone/s/figures

You may also use PLOS’s free figure tool, NAAS, to help you prepare publication quality figures: https://journals.plos.org/plosone/s/figures#loc-tools-for-figure-preparation.

NAAS will assess whether your figures meet our technical requirements by comparing each figure against our figure specifications.

# Author comment: Author response to Decision Letter 2

**DOI:** 10.1371/journal.pone.0358882.r004

24 Aug 2026

1. The values reported in the text are inconsistent with those shown in Figures 3 and 4. The authors should carefully verify all performance metrics and ensure consistency between the figures and the manuscript text. For example, in Line 508, according to Figure 3, 83.4% represents sensitivity, not specificity, whereas 50.8% represents precision, not sensitivity. The authors may find the following references useful for the appropriate calculation, application, and interpretation of these performance metrics: 10.1016/j.ipm.2009.03.002 and https://doi.org/10.3390/bioengineering11121250.

Response: We thank the reviewer for identifying this inaccuracy in our reporting. Precision, specificity and sensitivity is now correctly reported throughout the manuscript (Discussion lines 509, 510, 512, 515, 516, 528, 529, 530). We also added a sentence in the methods to state how these were calculated, Methods lines 334-338 “Per-class performance is reported via confusion matrix for the final model, with precision, sensitivity and specificity reported. For each class, sensitivity was calculated as TP/(TP+FN), precision as TP/(TP+FP), and specificity as TN/(TN+FP), where TP, TN, FP and FN denote true positives, true negatives, false positives and false negatives, respectively; metrics were calculated using a one-versus-rest approach”.

2. The authors should clarify how the accuracy values reported in Lines 424–425 were calculated.

Response: We thank the reviewer for identifying that the calculation and aggregation of the reported accuracy values was insufficiently described. We have revised the Methods and Results to clarify that accuracy was calculated at the stride-window level as the proportion of held-out windows correctly classified (Methods line 332-333 “Accuracy was calculated for each validation test set as the proportion of held-out stride windows correctly classified”). We now specify how mean accuracy, SD and 95% confidence intervals were calculated across validation iterations (Methods line 342-345 “The mean accuracy across the five test folds/groups are reported for both LSO and CV, with standard deviation (SD) calculated across fold/group accuracies and 95% confidence intervals (CI) calculated using the Student’s t distribution (±t_0.975,4×SD/√5)”). The reported values have been revised to add clarity to this (results lines 425-428 “the re-optimised model achieved a mean LSO accuracy across held-out participant groups of 56.89% (SD 5.5%; 95% CI 50.1–63.7%) and a mean CV accuracy of 83.64% (SD 0.8%; 95% CI 82.6–84.7%)” and lines 469-470 “and achieved a mean LSO accuracy across held-out participant groups of 61.56% (SD 16.1%; 95% CI 45.4-77.7%) and a mean CV accuracy of 82.90% (SD 2.1%; 95% CI 80.8-85.0%)”).

3. Figure 2 has no panel key. Please add a key mapping each panel to its terrain type.

Response: We thank the reviewer for identifying that Figure 2 does not have a panel key. A,B,C,D added to figure and key added to figure legend, Methods tine 274-275 “A) Uneven ground (cobble stones), B) Grass, C) Unstable gravel, D) Stairs.”.

4. The authors should conduct a thorough typographical and grammatical proofread of the manuscript. Examples include “prothesis” in Line 220 and “recorded” in Line 255.

Response: We thank the reviewer for identifying the errors in the manuscript. The manuscript has now been checked with mistakes corrected throughout the manuscript.

Attachment Submitted filename: response_to_reviewers_auresp_2.docx

# Peer review: Decision Letter 2

**DOI:** 10.1371/journal.pone.0358882.r005

**Authors:** Gary Guerra

7 Sep 2026

Lower limb prosthetic users’ activity classification through real-world inertial sensing

PONE-D-26-02026R2

Dear Dr. Wassall

We’re pleased to inform you that your manuscript has been judged scientifically suitable for publication and will be formally accepted for publication once it meets all outstanding technical requirements.

Within one week, you’ll receive an e-mail detailing the required amendments. When these have been addressed, you’ll receive a formal acceptance letter and your manuscript will be scheduled for publication.

An invoice will be generated when your article is formally accepted. Please note, if your institution has a publishing partnership with PLOS and your article meets the relevant criteria, all or part of your publication costs will be covered. Please make sure your user information is up-to-date by logging into Editorial Manager at Editorial Manager® and clicking the ‘Update My Information' link at the top of the page. For questions related to billing, please contact billing support.

If your institution or institutions have a press office, please notify them about your upcoming paper to help maximize its impact. If they’ll be preparing press materials, please inform our press team as soon as possible -- no later than 48 hours after receiving the formal acceptance. Your manuscript will remain under strict press embargo until 2 pm Eastern Time on the date of publication. For more information, please contact onepress@plos.org.

Kind regards,

Gary Guerra, Ph.D.

Academic Editor

PLOS One

# Editor report: Acceptance letter

**DOI:** 10.1371/journal.pone.0358882.r006

**Authors:** Gary Guerra

PONE-D-26-02026R2

PLOS One

Dear Dr. Wassall,

I'm pleased to inform you that your manuscript has been deemed suitable for publication in PLOS One. Congratulations! Your manuscript is now being handed over to our production team.

At this stage, our production department will prepare your paper for publication. This includes ensuring the following:

* All references, tables, and figures are properly cited

* All relevant supporting information is included in the manuscript submission,

* There are no issues that prevent the paper from being properly typeset

You will receive further instructions from the production team, including instructions on how to review your proof when it is ready. Please keep in mind that we are working through a large volume of accepted articles, so please give us a few days to review your paper and let you know the next and final steps.

Lastly, if your institution or institutions have a press office, please let them know about your upcoming paper now to help maximize its impact. If they'll be preparing press materials, please inform our press team within the next 48 hours. Your manuscript will remain under strict press embargo until 2 pm Eastern Time on the date of publication. For more information, please contact onepress@plos.org.

You will receive an invoice from PLOS for your publication fee after your manuscript has reached the completed accept phase. If you receive an email requesting payment before acceptance or for any other service, this may be a phishing scheme. Learn how to identify phishing emails and protect your accounts at https://explore.plos.org/phishing.

If we can help with anything else, please email us at customercare@plos.org.

Thank you for submitting your work to PLOS One and supporting open access.

Kind regards,

PLOS One Editorial Office Staff

on behalf of

Dr. Gary Guerra

Academic Editor

PLOS One
